# Mitochondrial Diseases: Molecular Pathogenesis and Therapeutic Advances

**DOI:** 10.1002/mco2.70385

**Published:** 2025-09-12

**Authors:** Jialun Mei, Peng Ding, Chuan Gao, Jian Zhou, Zhiwei Li, Changqing Zhang, Junjie Gao

**Affiliations:** ^1^ Department of Orthopaedics Shanghai Sixth People's Hospital Affiliated to Shanghai Jiao Tong University School of Medicine Shanghai China; ^2^ Institute of Microsurgery on Extremities and Department of Orthopedic Surgery Shanghai Sixth People's Hospital Affiliated to Shanghai Jiao Tong University School of Medicine Shanghai China; ^3^ Division of Hepatobiliary and Pancreatic Surgery Department of Surgery The First Affiliated Hospital Zhejiang University School of Medicine Hangzhou Zhejiang China

**Keywords:** mitochondrial gene editing, mitochondrial DNA (mtDNA), gene therapy, base editing, mitochondrial diseases, genetic medicine, therapeutic strategies

## Abstract

Mitochondrial diseases are a heterogeneous group of inherited disorders caused by pathogenic variants in mitochondrial DNA (mtDNA) or nuclear genes encoding mitochondrial proteins, culminating in defective oxidative phosphorylation and multisystem involvement. Key pathogenic mechanisms include heteroplasmy driven threshold effects, excess reactive oxygen species, disrupted mitochondrial dynamics and mitophagy, abnormal calcium signaling, and compromised mtDNA repair, which together cause tissue‐specific energy failure in high demand organs. Recent advances have expanded the therapeutic landscape. Precision mitochondrial genome editing—using mitochondrial zinc finger nucleases, mitochondrial transcription activator‐like effector nucleases, DddA‐derived cytosine base editor, and other base editing tools—enables targeted correction or rebalancing of mutant genomes, while highlighting challenges of delivery and off‐target effects. In parallel, metabolic modulators (e.g., coenzyme Q10, idebenone, EPI‐743) aim to restore bioenergetics, and mitochondrial replacement technologies and transplantation are being explored. Despite these promising strategies, major challenges remain, including off‐target effects, precise delivery, and ethical considerations. Addressing these issues through multidisciplinary research and clinical translation holds promise for transforming mitochondrial disease management and improving patient outcomes. By bridging the understanding of mitochondrial dysfunction with advanced therapeutic interventions, this review aims to shed light on effective solutions for managing these complex disorders.

## Graphical abstract

Key pathogenic mechanisms—OXPHOS defects, heteroplasmy, impaired mtDNA repair, disrupted dynamics/mitophagy, and inflammatory signaling—drive multiorgan involvement in mitochondrial diseases. Targeted interventions include metabolic drugs (CoQ10, idebenone, EPI‐743), mitochondrial gene editing (DdCBE, mitoBEs, CyDENT/mitoTALEN/mtZFN), and mitochondrial replacement/transplantation (restricted clinical use / largely preclinical). This graphic links mechanism to therapy and translational gaps.

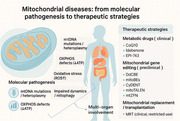

## 1 Introduction

Mitochondria, the powerhouses of eukaryotic cells, have long intrigued scientists due to their unique origin and critical role in cellular function. The prevailing endosymbiotic theory posits that mitochondria emerged from a symbiotic relationship between primitive eukaryotic cells and an ancestral bacterium [1, 2, 3, 4]. This arrangement allowed the bacterium to provide energy to the eukaryotic host through cellular respiration, while the host provided a stable environment [5]. Over time, this symbiotic partnership became so intimate that the bacterium evolved into an organelle within the host cell, giving rise to mitochondria [6].

The increasing prevalence and complexity of mitochondrial diseases have driven significant interest in understanding their molecular pathogenesis. Mitochondrial diseases often arise from point mutations, deletions, or duplications in mitochondrial DNA (mtDNA), which impair oxidative phosphorylation (OXPHOS) and adenosine triphosphate (ATP) production, thereby contributing to energy deficits in affected cells [7]. The severity of these diseases is often influenced by the level of heteroplasmy—the coexistence of normal and mutant mtDNA within cells [8]. In addition to mtDNA mutations, other key molecular mechanisms involved in mitochondrial diseases include oxidative stress, impaired mitochondrial quality control (e.g., mitophagy), disruptions in calcium signaling, and deficiencies in mtDNA repair mechanisms [9]. Oxidative stress, driven by increased reactive oxygen species (ROS), further damages mtDNA and mitochondrial proteins, creating a vicious cycle of dysfunction. Mitophagy, the process of selectively degrading damaged mitochondria, plays a crucial role in maintaining mitochondrial quality, and its dysregulation can exacerbate disease progression [10]. These molecular mechanisms, along with heteroplasmy, contribute to the intricate relationship between mtDNA mutations and mitochondrial dysfunction, which has been the focus of extensive research aiming to better understand the pathogenesis of these disorders.

To address these fundamental challenges, a deeper understanding of mitochondrial disease mechanisms is critical. Indeed, insights into mitochondrial dysfunction including oxidative stress, mtDNA repair deficits, and impaired mitochondrial quality control are not only key for clarifying disease pathology but also serve as essential requirement for developing effective therapeutic strategies. Recent advancements in molecular genetics and biotechnology have significantly expanded our therapeutic toolkit, with gene editing technologies emerging as particularly promising due to their potential to directly rectify pathogenic mutations in mtDNA. Novel mitochondrial genome editing methods, such as mitochondrial zinc finger nucleases (ZFNs), mitochondrial transcription activator‐like effector nucleases (mitoTALENs), and base editing tools like DddA‐derived cytosine base editor (DdCBE) and mtDNA base editors (mitoBEs), are already showing transformative potential in preclinical models. These techniques provide unique opportunities to correct or modulate pathogenic mutations without introducing double‐strand breaks (DSBs), thus reducing potential risks and side effects associated with traditional genome‐editing approaches.

However, therapeutic progress in mitochondrial diseases extends beyond gene editing alone. A comprehensive approach incorporating metabolic modulation, mitochondrial replacement techniques, and pharmacological interventions is also essential, as mitochondrial diseases typically exhibit complex phenotypes and affect multiple organ systems. Therefore, effective management likely requires integrated, multidisciplinary therapeutic strategies.

This review aims to bridge the gap between our evolving molecular understanding of mitochondrial diseases and the emerging spectrum of therapeutic possibilities. Specifically, we comprehensively explore the complex mechanisms underlying mitochondrial dysfunction including oxidative stress, heteroplasmy dynamics, impaired quality control systems, and mitochondrial gene homeostasis. Subsequently, we highlight current advancements and clinical implications of various therapeutic approaches, emphasizing gene editing techniques alongside other promising treatments. By systematically connecting disease mechanisms with recent therapeutic developments, we provide insights into the future of mitochondrial disease management, ultimately aiming to enhance clinical outcomes and improve the quality of life for patients with these challenging conditions.

## 2 Mitochondrial Fundamentals

**FIGURE 1:**
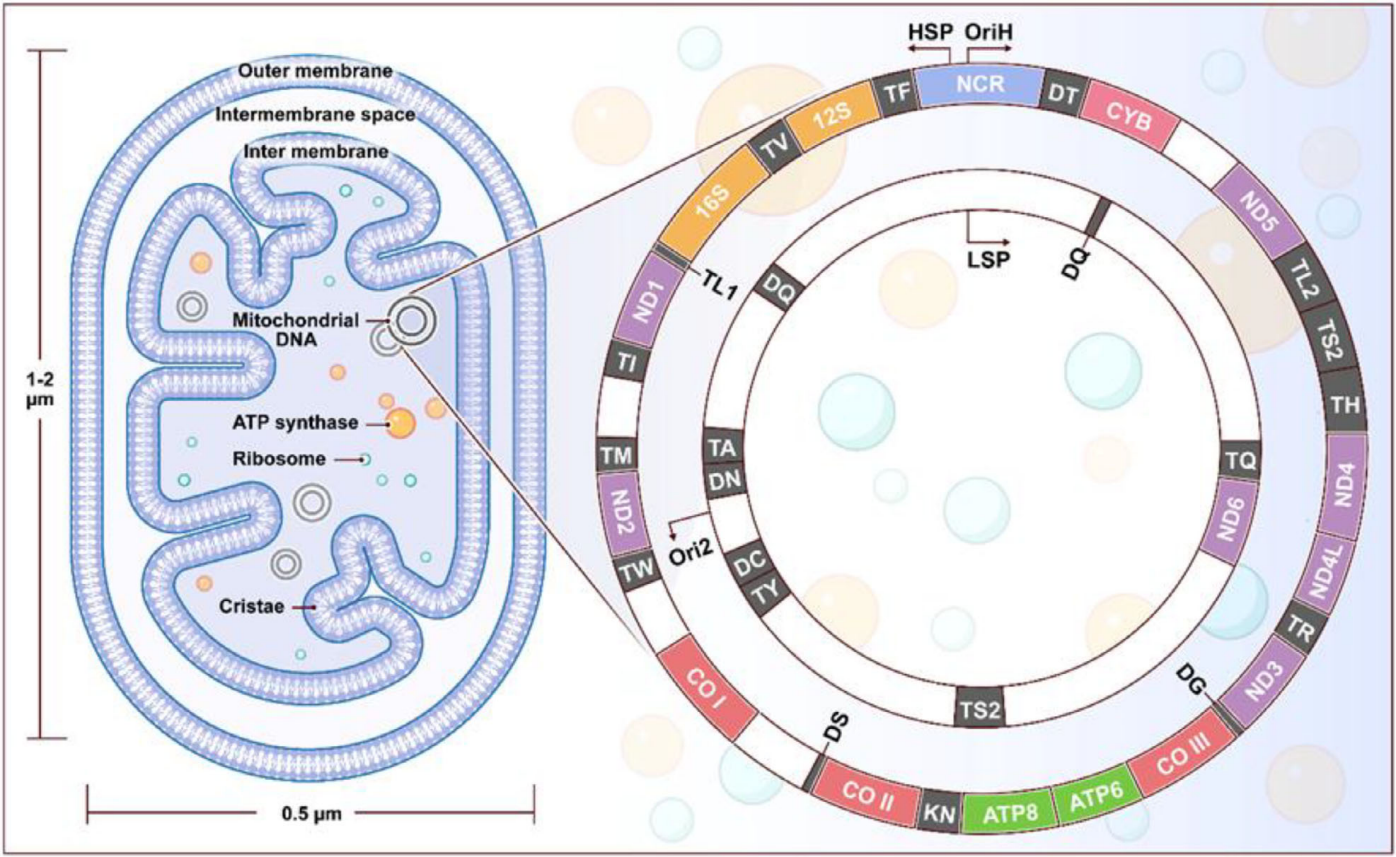
The mammalian mitochondrial genome: Mitochondria are small, double‐membraned organelles within cells, typically measuring 1–10 µm in size and often taking on elliptical or elongated cylindrical shapes. Inside mitochondria, the inner membrane is extensively folded into structures called cristae, which greatly increase its surface area. These cristae contain key components of the electron transport chain and the oxidative phosphorylation system, critical for generating cellular energy in the form of adenosine triphosphate (ATP). The mitochondrial matrix, located within the inner membrane, houses mitochondrial DNA, mitochondrial ribosomes, and machinery for protein synthesis, playing essential roles in various cellular processes. Mitochondrial DNA encodes essential components of the respiratory chain complexes I, III, and IV, as well as ATP synthase, all of which are vital for adenosine triphosphate (ATP) generation. Specifically, human mtDNA contains the genetic information for seven subunits of complex I (ND1–5, shown in purple), one subunit of complex III (CYB), three subunits of complex IV (COI–III, depicted in red), and two subunits of complex V (ATP8/6). Additionally, mtDNA encodes 22 transfer RNAs (tRNAs) and two ribosomal RNAs (rRNAs, represent in green), necessary for the translation of these mitochondrial proteins. This genetic information is densely packed and spans both DNA strands: a guanine‐rich heavy (H) strand and a cytosine‐rich light (L) strand. One major noncoding region (NCR) is present, housing the origin of heavy‐strand replication (OriH) and the promoters for heavy‐strand (HSP) and light‐strand (LSP) transcription. Another origin of replication dedicated to light‐strand synthesis (OriL) is situated approximately 11 kb away from OriH. The region between OriH and OriL is termed the major arc, while the smaller remaining region is referred to as the minor arc.

Mitochondria are double‐membrane bound organelles widely recognized as the “powerhouses” of the cell due to their critical role in ATP production through OXPHOS [11]. Their unique ultrastructure enables this function. The outer membrane is relatively permeable and contains porins that allow the diffusion of small solutes [11]. In contrast, the inner membrane is highly selective and extensively folded into cristae, significantly increasing the surface area available for hosting the protein complexes of the electron transport chain (ETC) [12]. The space enclosed by the inner membrane, known as the matrix, contains enzymes, mitochondrial ribosomes, and genetic material necessary for local transcription and translation [13] (Figure 1).

**FIGURE 2:**
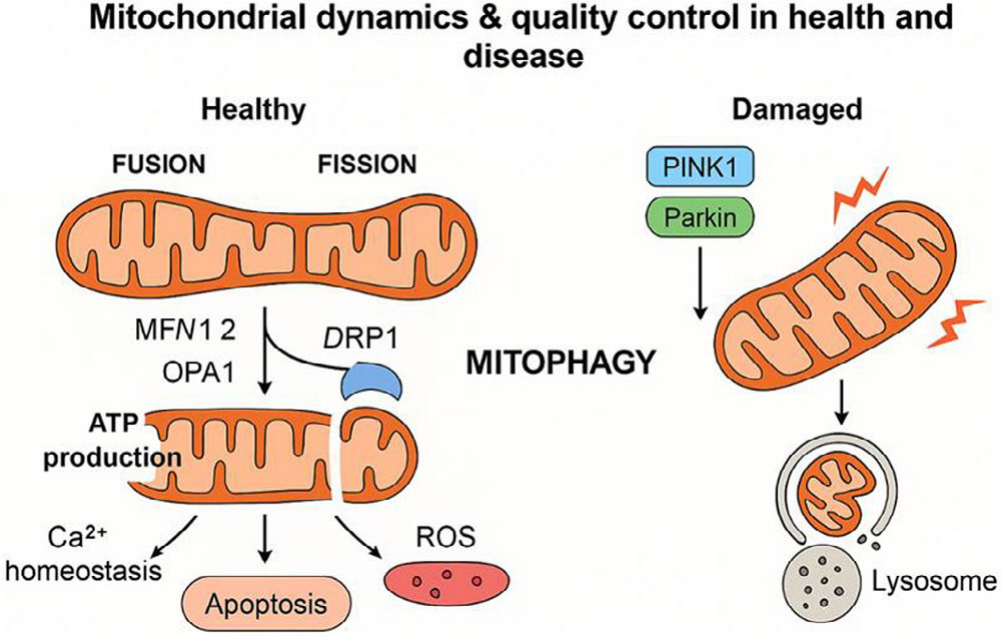
Mitochondrial dynamics and quality control in health and disease. Healthy mitochondria undergo continuous fusion (mediated by mitofusin 1/2 (MFN1/2) and optic atrophy protein 1 (OPA1) and fission (mediated by dynamin‐related protein 1 (DRP1) to maintain bioenergetic function, calcium (Ca^2^⁺) homeostasis, reactive oxygen species (ROS) balance, and regulation of apoptosis. Upon damage, PTEN‐induced putative kinase 1 (PINK1) accumulates on the outer mitochondrial membrane and recruits the E3 ubiquitin ligase Parkin, initiating selective mitochondrial autophagy (mitophagy). Damaged mitochondria are subsequently sequestered into autophagosomes and degraded in lysosomes, preserving mitochondrial quality and cellular health.

Functionally, mitochondria are indispensable for cellular metabolism and signaling. Beyond ATP synthesis, they regulate intracellular calcium homeostasis, modulate ROS, and mediate intrinsic apoptotic pathways [14]. In OXPHOS, mitochondria use electrons derived from nutrients to create a proton gradient across the inner membrane, ultimately driving ATP synthase activity [15]. Additionally, mitochondria serve as reservoirs of calcium ions and buffer cytosolic Ca^2^⁺, influencing various signal transduction cascades [16]. In the context of apoptosis, mitochondrial outer membrane permeabilization (MOMP) leads to the release of cytochrome *c* and other proapoptotic factors, triggering caspase activation and programmed cell death [17]. These processes collectively maintain mitochondrial integrity and cellular homeostasis (Figure 2).

A key distinction of mitochondria is that they possess their own genetic material, mtDNA, which is separate from the nuclear DNA (nucDNA) found in the nucleus. MtDNA is a small, circular molecule resembling bacterial DNA, while nucDNA is linear and significantly larger [18, 19]. This difference in genetic architecture extends to their coding systems. MtDNA encodes only 37 genes, crucial for OXPHOS, and lacks histone protection, making it more vulnerable to mutations caused by ROS [20]. In contrast, nucDNA is protected by histones and possesses a more elaborate repair system, making it more resilient to mutations [5]. MtDNA structural features include a porous outer membrane, which allows the free passage of small molecules, and an intricately folded inner membrane with cristae, which greatly increases the surface area for energy production. Human mtDNA, approximately 16.6 kb in length, encodes crucial components such as subunits of respiratory chain complexes (I, III, and IV), ATP synthetase, 22 transport RNAs (tRNAs), and 2 ribosomal RNAs (rRNAs) [6, 18, 21] (Figure 1).

Despite their distinct genomes, mitochondria and the nucleus maintain a tightly coordinated genetic relationship. While mtDNA encodes key components of the ETC, the majority of mitochondrial proteins are encoded by nuclear genes, synthesized in the cytosol, and imported posttranslationally into mitochondria [22]. This division of labor is the result of evolutionary gene transfer following the endosymbiotic origin of mitochondria [6]. Replication of mtDNA is carried out independently of the cell cycle and utilizes a dedicated DNA polymerase γ, distinct from nuclear replication machinery. Unlike nucDNA, which replicates during S phase in a tightly regulated manner, mtDNA replication can occur continuously, governed more by mitochondrial biogenesis signals than cell cycle checkpoints [23]. Additionally, mtDNA lacks introns and has polycistronic transcription units, in contrast to the highly structured and intron‐rich nature of nucDNA [24]. This streamlined architecture supports rapid transcription and translation, which is vital for energy‐demanding tissues.

## 3 Overview of Mitochondrial Diseases

Mitochondrial diseases constitute a diverse group of inherited disorders resulting from dysfunction in the mitochondrial respiratory chain [25]. They can be caused by mutations in either the mtDNA or nuclear genes that affect mitochondrial function. These conditions are among the most common groups of inherited metabolic diseases [26]. According to epidemiological studies such as Gorman et al. [25], the minimum prevalence of mitochondrial disease caused by mtDNA mutations is estimated at approximately one in 5000 individuals, although this number is likely underestimated due to diagnostic challenges. When nucDNA mutations affecting mitochondrial function are included, the prevalence rises further. Mitochondrial diseases may manifest at any age and affect multiple organs, with high‐energy‐demanding tissues such as the brain, skeletal muscle, and heart being especially vulnerable [27].

In recent years, studies of mtDNA have revealed its intricate connections to human diseases such as Leigh syndrome (LS), NARP syndrome (neuron, ataxia, and retinitis pigmentosa), and Leber hereditary optic neuropathy (LHON) [28, 29, 30, 31] (Table 1). The varying degrees of heteroplasmy within different tissues explain the diversity of symptoms seen in these conditions. For instance, LS often affects the central nervous system, leading to severe developmental and neurological issues [28, 29]. In contrast, LHON predominantly affects the optic nerves, causing vision loss [30, 31]. The clinical heterogeneity in these mitochondrial diseases can be attributed to the threshold effect of heteroplasmy, where different tissues tolerate different levels of mutant mtDNA before function is impaired.

**TABLE 1:** Signature mitochondrial syndromes at a glance.

Syndrome/abbreviation	Primary mutation(s)/gene(s)	Typical onset age	Main clinical spectrum	Key investigations	Common complications
Leigh syndrome (LS)	mtDNA: MT‐ATP6, MT‐ND genes; nDNA: SURF1, PDHA1, NDUFSx	Infancy/early childhood	Psychomotor regression, hypotonia, brainstem dysfunction	MRI: bilateral symmetric basal ganglia/brainstem lesions; lactate peak on MRS	Respiratory failure, feeding difficulties
MELAS (mitochondrial encephalomyopathy, lactic acidosis, and stroke‐like episodes)	m.3243A>G in MT‐TL1; other mt‐tRNA mutations	Childhood–young adult	Stroke‐like episodes, seizures, hearing loss, diabetes	MRI: cortical/subcortical lesions not confined to vascular territories; muscle biopsy: ragged‐red fibers	Cognitive decline, cardiomyopathy
MERRF (myoclonic epilepsy with ragged‐red fibers)	m.8344A>G in MT‐TK; other mt‐tRNA mutations	Childhood–adolescence	Myoclonus, generalized epilepsy, ataxia	Muscle biopsy: ragged‐red fibers; EEG: generalized spike‐wave	Hearing loss, optic atrophy
LHON (Leber hereditary optic neuropathy)	m.11778G>A (MT‐ND4), m.3460G>A (MT‐ND1), m.14484T>C (MT‐ND6)	Young adult males (typically 15–35 years)	Subacute painless central vision loss	Fundus: telangiectatic microangiopathy, swelling of retinal nerve fiber layer; OCT: RNFL thinning	Permanent visual impairment
Mitochondrial myopathies	Multiple mtDNA deletions (e.g., TK2, POLG), mt‐tRNA mutations	Variable	Progressive external ophthalmoplegia (PEO), ptosis, limb muscle weakness	Muscle biopsy: COX‐negative fibers; EMG: myopathic pattern	Dysphagia, respiratory weakness

Abbreviations: COX, cytochrome c oxidase; EEG, electroencephalography; EMG, electromyography; MRI, magnetic resonance imaging; MRS, magnetic resonance spectroscopy; OCT, optical coherence tomography; RNFL, retinal nerve fiber layer.

The disease spectrum of mitochondrial disorders is broad and clinically heterogeneous, with many syndromes defined by specific symptom combinations and associated genetic mutations (Table 1).

### 3.1 Leigh Syndrome

LS, also known as subacute necrotizing encephalomyelopathy, is a progressive and often fatal neurodegenerative disorder primarily affecting the central nervous system. It typically presents in infancy or early childhood with psychomotor regression, hypotonia, feeding difficulties, failure to thrive, and episodes of lactic acidosis. Neurological deterioration is rapid and often associated with characteristic bilateral symmetric lesions in the basal ganglia, thalamus, brainstem, and occasionally cerebellum, which can be detected via magnetic resonance imaging (MRI). Respiratory failure due to brainstem involvement is a common cause of death in pediatric patients [32].

Genetically, LS exhibits striking heterogeneity. It can arise from mutations in either mtDNA or nucDNA, with more than 75 distinct genes implicated. The most frequently associated mtDNA mutations involve the MT‐ATP6 gene (e.g., m.8993T>G) [33], while nuclear gene mutations include SURF1, NDUFS1, NDUFS7, and others encoding respiratory chain complex I and IV subunits or assembly factors [33]. Mutations in SURF1, in particular, are a leading nuclear cause of LS and impair cytochrome *c* oxidase (COX) activity. The disorder is typically inherited in an autosomal recessive or maternal (for mtDNA mutations) fashion, depending on the underlying genetic defect.

### 3.2 Mitochondrial Encephalopathy, Lactic Acidosis, and Stroke‐Like Episodes

Mitochondrial encephalopathy, lactic acidosis, and stroke‐like episodes (MELAS) syndrome is one of the most recognized and well‐characterized mitochondrial encephalomyopathies. It is defined by a triad of mitochondrial encephalopathy, lactic acidosis, and stroke‐like episodes. The onset typically occurs in childhood or early adulthood and presents with recurrent seizures, migraines, muscle weakness, vomiting, and acute neurological deficits resembling strokes—but these episodes do not follow typical vascular territories [34]. Progressive sensorineural hearing loss and diabetes mellitus are also commonly associated findings.

At the molecular level, MELAS is most frequently associated with a point mutation in the mitochondrial tRNA^Leu(UUR) gene, particularly the A3243G mutation [35], which accounts for approximately 80% of cases. This mutation impairs mitochondrial protein synthesis, leading to deficient respiratory chain complex activity (especially complexes I and IV) and impaired OXPHOS. The resulting energy deficit is especially detrimental to tissues with high metabolic demand such as the brain, skeletal muscle, and heart [35].

Pathologically, stroke‐like episodes in MELAS are thought to arise from abnormal mitochondrial angiopathy, mitochondrial proliferation in smooth muscle, and impaired vascular autoregulation, leading to regional cerebral hypoperfusion and neuronal injury [34]. MRI often reveals cortical and subcortical signal abnormalities that do not correspond to standard arterial distributions.

### 3.3 Myoclonic Epilepsy with Ragged Red Fibers

Myoclonic epilepsy with ragged red fibers (MERRF) is a rare but clinically distinct mitochondrial encephalomyopathy characterized by a constellation of neuromuscular symptoms. The hallmark features include myoclonic epilepsy, generalized seizures, ataxia, muscle weakness, and hearing loss. The disorder is named for the classic muscle biopsy finding of “ragged red fibers” (RRFs)—subsarcolemmal accumulation of abnormal mitochondria visible with Gomori trichrome staining [36].

Most cases of MERRF are associated with a maternally inherited mtDNA point mutation A8344G in the MT‐TK gene, which encodes mitochondrial tRNA^Lys [37]. This mutation impairs mitochondrial protein synthesis, compromising respiratory chain complex function (particularly complexes I and IV), and thus reducing ATP production. As in other mitochondrial disorders, the level of heteroplasmy—the ratio of mutated to wild‐type mtDNA—varies between tissues and contributes to the phenotypic variability observed even among family members.

Clinically, MERRF may begin in childhood or adolescence, with progressive neuromuscular decline. While myoclonus is the most prominent symptom, other neurological features such as dementia and pyramidal signs may appear later [36]. Some patients also show overlapping features with MELAS syndrome due to dual mutations or complex mtDNA rearrangements [36].

The diagnosis is supported by muscle biopsy, lactate elevation, MRI, EEG findings, and, most definitively, by molecular genetic testing for mtDNA mutations [36]. Importantly, not all individuals with the A8344G mutation show RRFs, emphasizing the need for molecular confirmation.

### 3.4 Leber Hereditary Optic Neuropathy

LHON is a maternally inherited mitochondrial disorder primarily affecting the optic nerves, resulting in subacute, painless, and often irreversible central vision loss, typically in young adult males. The vision impairment usually begins in one eye and progresses to the fellow eye within weeks to months. On fundoscopic examination, early features may include telangiectatic microangiopathy of the retinal nerve fiber layer and optic disc hyperemia [38].

LHON is caused by point mutations in mtDNA affecting subunits of complex I (NADH dehydrogenase) of the mitochondrial respiratory chain, leading to impaired ATP production and increased oxidative stress in retinal ganglion cells. The three primary mutations—m.11778G>A (ND4), m.3460G>A (ND1), and m.14484T>C (ND6)—account for over 90% of LHON cases worldwide. Among them, m.11778G>A is the most prevalent and often associated with a poor visual prognosis [39].

Unlike many other mitochondrial disorders, LHON shows a unique pattern of incomplete penetrance and marked gender bias: although the mutation is maternally inherited, less than 50% of male carriers and less than 10% of female carriers develop symptoms [38]. This phenomenon has been attributed to secondary environmental triggers (e.g., smoking, alcohol), mitochondrial haplogroups, and possibly X‐linked nuclear modifying genes [38].

### 3.5 Mitochondrial Myopathies

Mitochondrial myopathies represent a broad spectrum of neuromuscular disorders resulting from defects in mitochondrial OXPHOS. They are clinically characterized by muscle weakness, exercise intolerance, myalgia, and in some cases, systemic involvement such as ptosis, ophthalmoplegia, cardiomyopathy, or diabetes [40]. These myopathies may be isolated or part of multisystem mitochondrial syndromes such as Kearns–Sayre syndrome or progressive external ophthalmoplegia [41].

Pathogenetically, mitochondrial myopathies may be caused by mutations in mtDNA, such as large‐scale deletions or point mutations (e.g., in tRNA genes), or by nuclear gene mutations that disrupt mitochondrial protein synthesis, DNA replication, or respiratory complex assembly [40]. The resulting impairment in ATP production leads to energy failure in muscle fibers, particularly under conditions of increased metabolic demand.

Muscle biopsy is often diagnostic, revealing RRFs)∖ due to subsarcolemmal mitochondrial proliferation, COX negative fibers, or succinate dehydrogenase hyperactivity. Biochemical assays may show deficiencies in complexes I, III, IV, or V.

One hallmark of mitochondrial myopathies is the variable heteroplasmy—a coexistence of mutated and wild‐type mtDNA within cells—which leads to substantial phenotypic variability both between patients and among tissues of the same individual [42]. This heterogeneity poses a challenge for diagnosis and prognosis.

The wide‐ranging manifestations, variable age of onset, and often overlapping phenotypes make diagnosis and treatment of mitochondrial diseases particularly challenging. Ongoing research into heteroplasmy dynamics, mitonuclear interactions, and targeted gene therapies holds promise for more effective diagnostics and personalized treatments.

## 4 Molecular and Cellular Pathomechanisms of Mitochondrial Diseases

### 4.1 Types of Mitochondrial Gene Mutations and Their Pathogenic Mechanisms

Mitochondrial genes, like all genetic material, are susceptible to damage caused by various factors, including oxidative stress, replication errors, and external sources such as radiation [43, 44]. Despite the multicopy nature of mtDNA, which provides some resilience against mutations by allowing the presence of intact copies, damage to these genes can still have profound consequences for cellular function if enough copies are affected [45]. To counteract this damage, mitochondria possess repair mechanisms, although they differ from those in the nuclear genome [46]. MtDNA mutations are often maternally inherited, leading to various mitochondrial diseases. These diseases result from heteroplasmy, a condition where both normal and mutant mtDNA coexist in cells. The severity of the disease often correlates with the proportion of mutant mtDNA. Despite the limited number of genes encoded by mtDNA, mutations in these genes can cause devastating disorders that affect high‐energy tissues, such as muscles and the nervous system [4]. Given the critical role of mitochondria in energy production and cellular homeostasis, there is an urgent need to develop efficient mtDNA editing technologies.

Mitochondrial diseases often arise from point mutations, deletions, or duplications in mtDNA, leading to impaired OXPHOS and ATP production. In recent years, scientists identified a novel mutation, m.8561C>G in MT‐ATP6/8, which causes a spectrum of mitochondrial symptoms, including ataxia, neuropathy, diabetes mellitus, and hormonal imbalances [47]. Such mutations exemplify how mtDNA alterations can result in multisystemic impacts, especially in organs with high energy demands. Additionally, other scientists described a specific 2 bp deletion in the mitochondrial ATP6 gene responsible for NARP syndrome (neuropathy, ataxia, and retinitis pigmentosa) [48], further emphasizing the devastating effects of mtDNA mutations on neuromuscular function. Recent studies have expanded on these findings by highlighting how these mutations not only impair ATP production but also disrupt calcium signaling, ROS balance, and apoptosis regulation, contributing to complex disease phenotypes [49]. These examples illustrate the intricate interplay between mtDNA mutations and cellular metabolic pathways, which ultimately lead to varying degrees of disease severity depending on mutation type and heteroplasmy levels.

### 4.2 Heteroplasmy and Disease Severity

Recent studies have shown the intricate relationship between mitochondrial heteroplasmy and disease progression. Gupta et al. [50] elucidated the nuclear genetic control of mtDNA copy number and heteroplasmy, highlighting the role of nuclear factors in determining mtDNA dynamics and heteroplasmic levels. This control is crucial, as the proportion of mutant mtDNA must exceed a specific threshold to manifest disease symptoms. Stewart and Chinnery [51] discussed the extreme heterogeneity of mtDNA across different human tissues, providing insights into why certain tissues, such as the brain and skeletal muscle, are more susceptible to mitochondrial dysfunction. These findings underscore the importance of tissue‐specific heteroplasmy levels in determining the severity and type of mitochondrial diseases.

Heteroplasmy denotes the coexistence of both normal and mutant mtDNA molecules within cells, and the severity of clinical symptoms often correlates with the proportion of mutant mtDNA, with symptoms becoming apparent when mutant mtDNA surpasses a threshold, typically approximately 60–90% of the mitochondrial population [52]. Furthermore, the clonal expansion of mutant mtDNA within postmitotic tissues further complicates disease progression [51]. In contrast, homoplasmy signifies the exclusive presence of either normal or mutant mtDNA within a cell, although even cells with homoplasmic mutations may not consistently exhibit clinical abnormalities [53].

### 4.3 Mitochondrial Inheritance Mechanisms and Phenotypic Variability

Mitochondrial inheritance mechanisms play a significant role in determining the phenotypic variability of mitochondrial diseases [54]. The maternal inheritance of mtDNA means that mutations are passed down through the maternal line, leading to a wide range of potential clinical outcomes depending on the level of heteroplasmy [54]. The interplay between mtDNA and nucDNA also contributes to phenotypic variability, as nuclear‐encoded proteins are crucial for mitochondrial function, including replication, repair, and energy production. Differences in nuclear gene variants can influence how cells cope with mitochondrial dysfunction, leading to variability in disease presentation even among individuals carrying the same mtDNA mutations [55].

Recent findings have highlighted the role of mitophagy in the context of mitochondrial diseases. Cao et al. [56] discovered that a mitochondrial SCF–FBXL4 ubiquitin E3 ligase complex can degrade mitophagy receptors such as BNIP3 and NIX, thus preventing excessive mitophagy that could contribute to mitochondrial dysfunction. This new insight helps explain the regulation of mitochondrial quality control and suggests potential therapeutic strategies to manipulate mitophagy for disease treatment. Mitophagy acts as a double‐edged sword; while it serves to remove damaged mitochondria and maintain cellular health, its dysregulation can exacerbate mitochondrial disease progression. Expanded discussion on the role of mitophagy includes recent discoveries on how proteins like FBXL4 regulate mitophagy to maintain mitochondrial health. Dysregulation of this process can lead to the accumulation of dysfunctional mitochondria, exacerbating mitochondrial disease symptoms [57]. Mitophagy acts as a double‐edged sword; while it serves to remove damaged mitochondria and maintain cellular health, its dysregulation can exacerbate mitochondrial disease progression [45]. Therefore, understanding how mitochondrial proteins like FBXL4 control mitophagy is critical for developing effective therapeutic strategies [56]. Given the maternal inheritance pattern of mtDNA mutations and the heteroplasmy threshold effect, developing gene editing technologies capable of selectively eliminating or correcting mutated mtDNA has become a key strategy for breaking the chain of disease transmission.

### 4.4 Mitochondrial Gene Homeostasis and Disease

mtDNA repair primarily involves base excision repair (BER) and single‐strand break repair pathways [46, 58], which are responsible for rectifying oxidative DNA lesions, alkylation, and deamination damage. Notably, while the presence of efficient repair mechanisms in mitochondria is acknowledged, the repair of DSBs, a more severe form of damage [59], remains a subject of debate. Unlike the nuclear genome, where DSBs typically stimulate recombination and repair, mtDNA DSBs often lead to the degradation of mtDNA molecules rather than successful repair [59, 60]. Low‐level nonhomologous end joining (NHEJ) activity in mitochondria has been suggested, but it appears to be relatively inefficient compared with other repair mechanisms [60]. As a result, the repair of DSBs in mitochondrial genes remains a complex and less understood aspect of mitochondrial genetics, with ongoing research aimed at unraveling these intricate processes and their implications for mitochondrial health and function.

In the context of mitochondrial gene homeostasis, it is crucial to recognize that these repair mechanisms play a pivotal role in mitigating damage to mitochondrial genes. This is particularly relevant because mitochondrial diseases encompass a wide range of disorders primarily arising from impaired mitochondrial function that profoundly disrupt cellular energy production [61]. These conditions stem from genetic abnormalities within two vital components of the mitochondrial genome: mtDNA and nucDNA [52, 62]. Understanding the maintenance of mitochondrial gene integrity is essential for comprehending how disruptions in these processes contribute to disease development.

### 4.5 Untargeted Genetic Manipulation of mtDNA for Disease Research

Due to the difficulties associated with site‐specific modifications of mtDNA, researchers have developed various methods for inducing random mutations. One method relies on chemical or radiation‐induced mutagenesis, coupled with a reduction in mtDNA copy numbers and negative selection to generate mutant cell lines [63]. These methods have resulted in the generation of several novel mtDNA mutant mammalian cell lines, which have been used to study fundamental mitochondrial processes and, in one instance, to produce embryonic stem cells for oocyte injection in the process of mouse model generation [64]. However, these experiments have also revealed that animal mtDNA is highly resistant to chemical‐ or radiation‐induced mutagenesis, presenting a significant challenge for researchers seeking to induce mutations in a controlled and targeted manner [65].

Advancements in genetic engineering have allowed for the introduction of random mutations in mtDNA by altering the mitochondrial replication machinery. One such breakthrough was the creation of the mtDNA mutator mouse, which carries a mutation that disrupts the proofreading activity of the catalytic subunit of the mtDNA replicase Pol γ (Polg1, D257A) [66]. These mutant mice have a progeria‐like phenotype and a reduced lifespan due to the accumulation of high levels of mtDNA mutations [66, 67]. By limiting the number of transmitted mutations, researchers were able to selectively breed mouse lines with clonally expanded heteroplasmic point mutations. One of these lines contained a heteroplasmic mt‐tRNA^Ala^ mutation, m.5024C>T, which led to the development of hypertrophic cardiomyopathy and associated cardiac defects in adulthood, a decrease in mt‐tRNA^Ala^ levels and impaired mitochondrial translation [68]. This mutation is equivalent to the human pathogenic m.5650G>A substitution, which causes mitochondrial myopathy [69, 70]. This mouse model is clinically relevant to many mtDNA diseases caused by mutations of mitochondrial tRNAs, which account for over 50% of mitochondrial disease patients [71]. The same method was also used in vitro to generate a collection of mouse cell lines for potential use in the generation of transmitochondrial mice [72]. Additionally, a fruit fly model was created using a proofreading‐deficient allele of PolG1, and an alternative model was generated based on the mitochondrially targeted, inducible cytidine deaminase APOBEC1 [73, 74]. While mtDNA mutator flies did not exhibit major phenotypes or a reduced lifespan, the mito‐APOBEC1 model led to increased C‐T (G‐A) mtDNA mutagenesis, limiting mitochondrial function, organismal vitality, and lifespan [75]. Comparison of mutation spectra between the PolG1 D263A and mito‐APOBEC1 models revealed that the quality of the mtDNA mutation profile was crucial for perturbing organismal fitness, rather than the mutation load. The mito‐APOBEC1 approach has potential for use in mammalian models, providing advantages over existing random mtDNA mutagenesis systems.

### 4.6 Bioenergetic and Cellular Signaling Dysfunction in Mitochondrial Diseases

#### 4.6.1 Bioenergetic Failure

Bioenergetic failure is a central pathological feature of mitochondrial diseases, arising from impaired OXPHOS III and reduced ATP production. Mitochondria generate over 90% of cellular ATP via the ETC and the chemiosmotic gradient established across the inner membrane [76]. In mitochondrial disorders, mutations in mtDNA or nuclear genes encoding ETC components or assembly factors compromise this system, leading to an insufficient energy supply, particularly in tissues with high metabolic demands such as the brain, heart, and skeletal muscle [77].

Defective OXPHOS function disrupts the mitochondrial proton gradient, diminishing the capacity of ATP synthase to phosphorylate ADP. This ATP shortfall impairs essential processes like ion transport, synaptic transmission, and muscle contraction. To compensate, affected cells may increase glycolytic flux via AMPK signaling, as shown in fibroblasts from patients with mitochondrial encephalomyopathy [78]. However, this metabolic shift is often insufficient and leads to an overaccumulation of lactate, a hallmark of mitochondrial dysfunction manifesting clinically as lactic acidosis [78].

Furthermore, secondary disruptions in NAD⁺/NADH balance, TCA cycle stalling, and coenzyme Q10 (CoQ) deficiency amplify the bioenergetic crisis. The inflexibility of metabolic adaptation in mitochondrial disease limits cellular resilience, ultimately leading to bioenergetic collapse, energy crisis, and cell death [76].

#### 4.6.2 Oxidative Stress and ROS Signaling

Oxidative stress is a major pathological driver in mitochondrial diseases, emerging from the overproduction of ROS during impaired OXPHOS. While ROS are natural byproducts of electron transport, their excessive accumulation—especially from dysfunctional complexes I and III—leads to damage of mtDNA, proteins, and lipids. This self‐amplifying cycle exacerbates mitochondrial dysfunction and promotes further ROS release, ultimately destabilizing redox homeostasis [79].

In mitochondrial diseases, defective antioxidant defenses such as glutathione or superoxide dismutase result in sustained oxidative stress, triggering both intrinsic apoptosis and proinflammatory signaling. Mitochondria‐generated ROS activate downstream pathways including NF‐κB, MAPKs, and p53, linking mitochondrial dysfunction to systemic inflammation and cell death [80]. Moreover, oxidative modifications to mtDNA often escape repair due to deficient mitochondrial BER systems, further aggravating mutation load [81].

Importantly, ROS are not solely destructive; they also act as signaling molecules regulating metabolic adaptation and stress responses. However, in disease states, this signaling becomes maladaptive, contributing to neuronal degeneration, myopathy, and multisystemic failure. Therapeutically, targeting mitochondrial ROS with agents such as MitoQ or SS‐31 shows promise in restoring redox balance in preclinical models [82].

#### 4.6.3 Mitochondrial Dynamics and Quality Control

Mitochondrial dynamics—the constant fusion and fission of mitochondria—are essential for maintaining mitochondrial function and integrity. These processes allow cells to segregate damaged mitochondria, facilitate mitochondrial biogenesis, and adjust morphology in response to metabolic demand. Disruptions in fusion or fission compromise bioenergetic capacity, enhance ROS production, and accelerate mtDNA instability [83, 84].

Fusion is predominantly mediated by mitofusins (MFN1/2) on the outer mitochondrial membrane and OPA1 on the inner membrane. Deficiencies in these proteins impair the mixing of mitochondrial contents and accelerate mtDNA depletion syndromes. Conversely, fission is primarily orchestrated by DRP1, a GTPase recruited to mitochondrial constriction sites. Excessive fission is associated with mitochondrial fragmentation and impaired ATP production. Studies have shown that pathogenic variants in DRP1, OPA1, and associated regulators such as FBXL4 contribute to various inherited mitochondrial disorders [85].

Mitophagy—selective autophagic removal of damaged mitochondria—is tightly linked to mitochondrial dynamics. The PINK1/Parkin pathway facilitates the recognition of depolarized mitochondria and recruits autophagy machinery for degradation. However, excessive mitophagy, especially in the absence of regulatory control by proteins like FBXL4, can lead to mitochondrial depletion and cellular dysfunction [85]. Recent studies show that FBXL4 stabilizes fusion‐promoting factors and prevents DRP1 overactivation, suggesting a central role in mitochondrial quality control [86].

Dysregulation of any component in this dynamic network—fusion, fission, or mitophagy—can result in mitochondrial disease, neurodegeneration, or myopathy. Restoring balanced dynamics has emerged as a promising therapeutic approach.

#### 4.6.4 Crosstalk with Apoptotic and Immune Pathways

Mitochondria are key arbiters of cellular fate through their regulation of apoptosis and immune signaling. In the intrinsic (mitochondrial) apoptotic pathway, stress signals such as DNA damage or calcium overload induce MOMP, mediated by proapoptotic BCL‐2 family proteins (e.g., BAX, BAK). This event leads to the release of cytochrome *c* into the cytosol, triggering apoptosome formation and caspase‐9 activation—a cascade that ultimately results in programmed cell death [87, 88].

Beyond cell death, mitochondria engage innate immunity by releasing damage‐associated molecular patterns, particularly mtDNA, which acts as an immunostimulatory signal. Cytosolic leakage of oxidized mtDNA activates pattern recognition receptors such as Toll‐like receptor 9 and the cGAS–STING pathway, inducing type I interferon and NF‐κB‐dependent inflammatory responses. This immune signaling has been directly implicated in the pathology of neurodegenerative and mitochondrial diseases [89, 90].

Interestingly, apoptosis and immune activation are not mutually exclusive. In “caspase‐suppressed apoptosis,” mtDNA can be extruded into the cytosol without cell lysis, thus decoupling death from inflammation. This condition amplifies immunopathology and may explain the sterile inflammation observed in mitochondrial encephalopathies and cardiomyopathies [91]. Targeting these mitochondria‐induced immune mechanisms—such as STING inhibitors or autophagy enhancers—emerges as a therapeutic frontier in dampening mitochondrial inflammation.

## 5 Therapeutic Advances: From Bench to Bedside

### 5.1 Small‐Molecule Therapies

Small‐molecule drugs offer a promising avenue for treating mitochondrial diseases by targeting metabolic dysfunctions without the delivery complexities of gene therapy. These compounds are typically orally bioavailable and systemically distributed, offering practical advantages in clinical contexts. Several small molecules have been studied in preclinical and clinical settings, including idebenone, EPI‐743 (vatiquinone), and bezafibrate, each targeting distinct aspects of mitochondrial pathophysiology.

Idebenone, a synthetic CoQ analog, enhances electron flux through the mitochondrial respiratory chain by transferring electrons directly to complex III, bypassing dysfunctional complex I activity. This mechanism has shown benefit in conditions like LHON. The RHODOS trial demonstrated that idebenone stabilized or improved visual acuity in over 50% of treated LHON patients versus 20% in placebo (*p* = 0.005) [92]. While idebenone has been approved by the European Medicines Agency for LHON, its application in other disorders like Friedreich's ataxia has met limitations; a Phase III trial failed to meet endpoints (NCT00537680) [93].

EPI‐743 (vatiquinone) is a para‐benzoquinone derivative that targets oxidative stress by modulating NADPH‐dependent oxidoreductases and enhancing glutathione biosynthesis. In a Phase II open‐label trial (NCT01721733), EPI‐743 improved survival and neurological outcomes in patients with LS, particularly in early‐onset forms [94]. However, a planned Phase III study was discontinued due to insufficient enrollment, limiting further evaluation [95]. Nonetheless, preclinical studies support its neuroprotective effects through modulation of redox homeostasis [95].

Bezafibrate, a pan‐PPAR agonist, enhances mitochondrial biogenesis by activating PGC‐1α‐dependent transcriptional pathways. In vitro and mouse models of mitochondrial myopathy have demonstrated that bezafibrate increases ATP production and fatty acid oxidation [96]. An open‐label trial in patients with mitochondrial myopathy reported improved exercise tolerance and reduced lactate levels [97], but large‐scale randomized controlled trials remain lacking.

While not a targeted small molecule, riboflavin (vitamin B2) has been shown to support ETC function by stabilizing flavoproteins in complexes I and II. Riboflavin supplementation has been especially effective in riboflavin‐responsive multiple acyl‐CoA dehydrogenase deficiency and certain complex I deficiencies [98]. However, evidence in common syndromic mitochondrial disorders like MELAS or LHON remains anecdotal or derived from small series.

Despite encouraging preclinical data, small‐molecule therapies face several limitations. These include variable bioavailability, lack of tissue specificity, and patient‐to‐patient heterogeneity due to differences in mtDNA mutation burden and heteroplasmy levels. To overcome these challenges, recent advances have explored nanoparticle‐mediated delivery platforms to selectively target mitochondria and enhance drug uptake [99].

### 5.2 Gene Therapy

#### 5.2.1 Mitochondrial Gene Editing Technology for Mitochondrial Diseases with Nucleases

**FIGURE 3:**
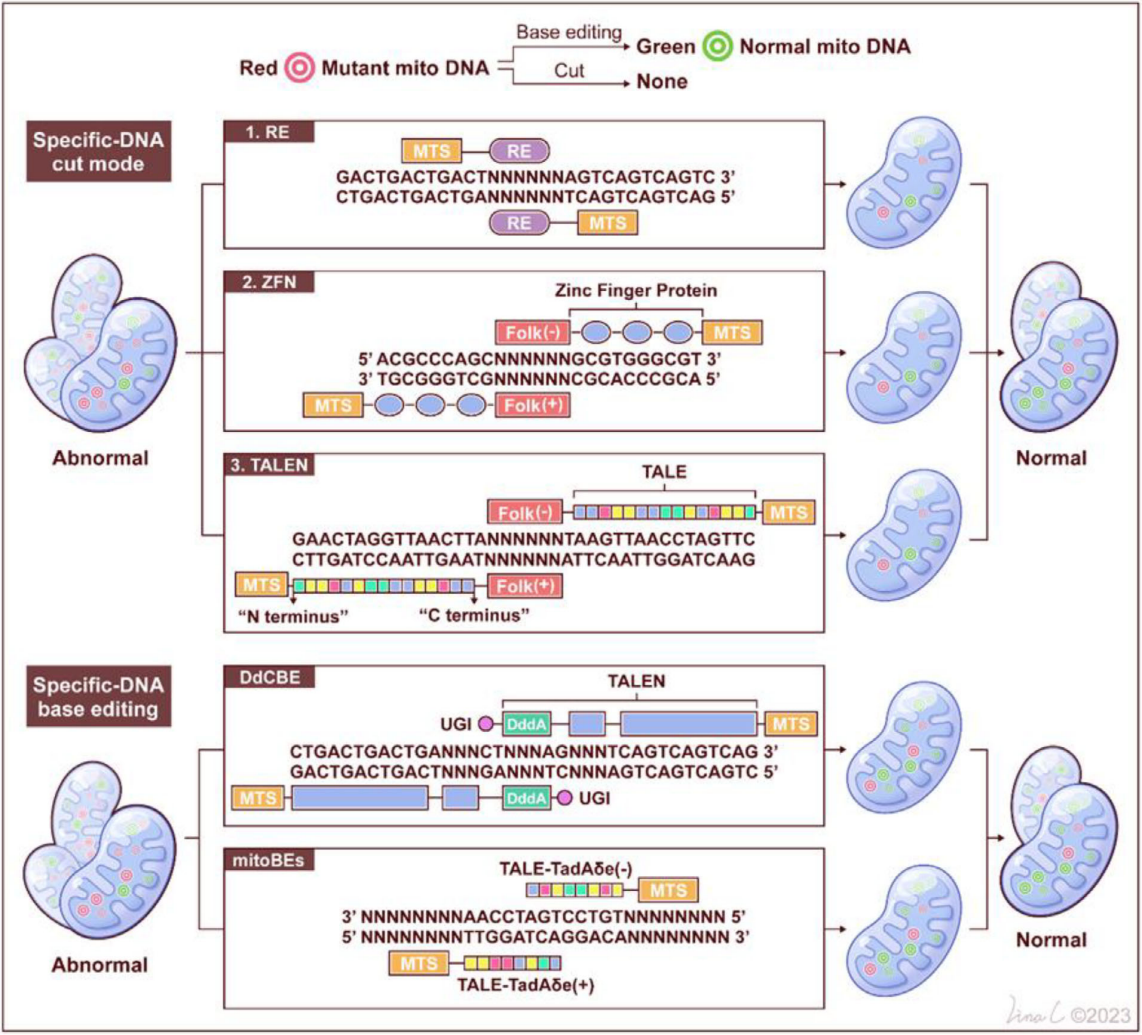
Overview of mitochondrial genome‐engineering strategies: Panel (A) illustrates the distinctive structure and functioning mechanism of mitochondrially targeted nucleases, which include restriction endonucleases (mitoRE), zinc finger nucleases (mtZFNs), and transcriptional activator‐like effectors nucleases (mitoTALENs). Within cells containing mutant mtDNA, these nucleases selectively identify mutated sequences (depicted in red). The induction of double‐strand breaks initiates the rapid degradation of the mutated mtDNA, leading to a reduction in the number of mutant copies. To restore the mtDNA copy count, the majority of the remaining mtDNA is of the wild‐type variety (depicted in blue). Consequently, this process replenishes the cells with normal mtDNA copies, ultimately enhancing the functionality of OXPHOS. While panel (B) illustrates the distinctive structure and functioning mechanism of base editors, which include DddA‐derived cytosine base editors (DdCBEs) and mtDNA base editors (mitoBEs). In cells containing a mixture of mutant and normal mt‐RNA, the base editor progressively rectifies the mutant mtDNA until the level of heteroplasmy falls below the threshold of pathogenicity.

Another promising avenue for addressing mitochondrial diseases involves gene editing, a powerful approach that aims to target and rectify mutated mitochondrial genes, thereby reducing the burden of mutant mtDNA and enhancing mitochondrial function. Unlike transplantation, gene editing offers a broader spectrum of applications. While often referred to as gene editing, it is important to note that the primary methods employed, such as restriction endonuclease (RE) technology, ZFN technology, and TALEN technology, primarily focus on the removal or modification of mutated mtDNA rather than precise editing. Despite this distinction, these innovative techniques hold the potential to revolutionize the field of mitochondrial medicine (Figure 3). These innovative techniques have the potential to revolutionize the field of mitochondrial medicine (Table 2).

**TABLE 2:** Pathogenic mitochondrial mutations confirmed with MITOMAP [172] and there potential curing methods. Most pathogenic point mutations can potentially be corrected or modeled using mitochondrial gene editing technology.

Locus	Allele	Position	Nucleotide change	Disease	Potential treatment
MT‐ATP6	m.8851T>C	8851	T‐C	BSN/Leigh syndrome	DdCBE/MitoBEs
	m.8969G>A	8969	G‐A	MLASA/IgG nephropathy	DdCBE/MitoBEs
	m.8993T>C	8993	T‐C	NARP/Leigh disease/MILS/other	DdCBE/MitoBEs
	m.8993T>G	8993	T‐G	NARP/Leigh disease/MILS/other	MitoRE/mitoZFN
	m.9035T>C	9035	T‐C	Ataxia syndromes	DdCBE/MitoBEs
	m.9155A>G	9155	A‐G	MIDD, renal insufficiency	DdCBE/MitoBEs
	m.9176T>C	9176	T‐C	FBSN/Leigh disease/spinocerebellar ataxia	DdCBE/MitoBEs
	m.9176T>G	9176	T‐G	Leigh disease/spastic paraplegia/spinocerebellar ataxia	MitoTALEN
	m.9185T>C	9185	T‐C	Leigh disease/ataxia syndromes/NARP‐like disease	DdCBE/MitoBEs
	m.9191T>C	9191	T‐C	Leigh disease	DdCBE/MitoBEs
	m.9205_9206del	9205	TA‐del	Encephalopathy/seizures/lacticacidemia	Not detected
MT‐ATP8/6	m.8528T>C	8528	T‐C	Infantile cardiomyopathy/hyperammonemia	DdCBE/MitoBEs
MT‐KN	m.8344A>G	8344	A‐G	MERRF	DdCBE/MitoBEs/mitoTALEN
MT‐CO1	m.7445A>G	7445	A‐G	SNHL	DdCBE/MitoBEs
MT‐CYB	m.14849T>C	14849	T‐C	EXIT/septo‐optic dysplasia	DdCBE/MitoBEs
MT‐CYB	m.15579A>G	15579	A‐G	Multisystem disorder, EXIT	DdCBE/MitoBEs
MT‐TL1	m.3243A>G	3243	A‐G	MELAS/MIDD/cardiomyopathy	DdCBE/MitoBEs/mitoTALEN
MT‐ND1	m.3376G>A	3376	G‐A	LHON MELAS overlap	DdCBE/MitoBEs
	m.3460G>A	3460	G‐A	LHON	DdCBE/MitoBEs
	m.3635G>A	3635	G‐A	LHON	DdCBE/MitoBEs
	m.3697G>A	3697	G‐A	MELAS/Leigh syndrome/LDYT/BSN	DdCBE/MitoBEs
	m.3700G>A	3700	G‐A	LHON	DdCBE/MitoBEs
	m.3733G>A	3733	G‐A	LHON	DdCBE/MitoBEs
	m.3890G>A	3890	G‐A	Progressive encephalomyopathy/Leigh syndrome/optic atrophy	DdCBE/MitoBEs
	m.3902_3908inv	3902	ACCTTGC‐GCAAGGT	EXIT+myalgia/severe LA+cardiac	DdCBE/MitoBEs
	m.4171C>A	4171	C‐A	LHON/Leigh‐like phenotype	DdCBE/MitoBEs
MT‐ND3	m.10158T>C	10158	T‐C	Leigh disease/MELAS	DdCBE/MitoBEs
	m.10191T>C	10191	T‐C	Leigh disease/Leigh‐like disease/ESOC	DdCBE/MitoBEs
	m.10197G>A	10197	G‐A	Leigh disease/dystonia/stroke/LDYT	DdCBE/MitoBEs
MT‐ND4	m.11777C>A	11777	C‐A	Leigh disease	Not detected
	m.11778G>A	11778	G‐A	LHON/progressive dystonia	DdCBE/MitoBEs
MT‐ND4L	m.10663T>C	10663	T‐C	LHON	DdCBE/MitoBEs
MT‐ND5	m.12706T>C	12706	T‐C	Leigh disease	DdCBE/MitoBEs
	m.13042G>A	13042	G‐A	Optic neuropathy/retinopathy/LD	DdCBE/MitoBEs
	m.13051G>A	13051	G‐A	LHON	DdCBE/MitoBEs
	m.13094T>C	13094	T‐C	Ataxia+PEO/MELAS/LD/LHON	DdCBE/MitoBEs
	m.13379A>G	13379	A‐G	LHON	DdCBE/MitoBEs
	m.13513G>A	13513	G‐A	Leigh disease/MELAS/LHON‐MELAS overlap syndrome	DdCBE/MitoBEs
	m.13514A>G	13514	A‐G	Leigh disease/MELAS/Ca^2+^ downregulation	DdCBE/MitoBEs/mitoTALEN
MT‐ND6	m.14453G>A	14453	G‐A	MELAS/Leigh disease	DdCBE/MitoBEs
	m.14459G>A	14459	G‐A	LDYT/Leigh disease/dystonia/carotid atherosclerosis risk	DdCBE/MitoBEs
	m.14482C>A	14482	C‐A	LHON	Not detected
	m.14482C>G	14482	C‐G	LHON	Not detected
	m.14484T>C	14484	T‐C	LHON	DdCBE/MitoBEs
	m.14487T>C	14487	T‐C	Dystonia/Leigh disease/ataxia/ptosis/epilepsy	DdCBE/MitoBEs
	m.14495A>G	14495	A‐G	LHON	DdCBE/MitoBEs
	m.14568C>T	14568	C‐T	LHON	DdCBE/MitoBEs

Abbreviations: BSN, basal ganglia necrosis; DdCBE, DddA‐derived cytosine base editor; ESOC, early‐onset cerebellar ataxia; EXIT, exercise intolerance; FBSN, fatal infantile basal ganglia disease; LD, Leigh disease; LDYT, Leber's hereditary optic neuropathy with dystonia; LHON, Leber hereditary optic neuropathy; MELAS, mitochondrial encephalomyopathy, lactic acidosis, and stroke‐like episodes; MERRF, myoclonic epilepsy with ragged‐red fibers; MIDD, maternally inherited diabetes and deafness; MILS, maternally inherited Leigh syndrome; mito‐RE, mitochondrial restriction endonuclease; MitoBEs, mitochondrial base editors; mitoTALEN, mitochondrial transcription activator‐like effector nuclease; mitoZFN, mitochondrial zinc finger nuclease; MLASA, myopathy, lactic acidosis, and sideroblastic anemia; mtDNA, mitochondrial DNA; NARP, neuropathy, ataxia, and retinitis pigmentosa; PEO, progressive external ophthalmoplegia; SNHL, sensorineural hearing loss.

##### 5.2.1.1 Restriction Endonuclease

Among the available methods for mitochondrial gene editing, REs have played a pivotal role. These enzymes are recognized for their ability to bind to specific DNA sequences and cleave phosphodiester bonds at precise sites on both DNA strands. REs offer several advantages, including accurate sequence recognition, high cutting efficiency, and site‐specific cleavage, making them valuable tools for gene editing. However, they have inherent limitations, such as their ability to recognize only a restricted set of nucleic acid sequences and their inability to be custom‐designed for specific applications [100].

REs have a straightforward operational process and were first utilized in mitochondrial gene editing [101]. The modified RE *Sma*I, with added mitochondrial targeting signals (MTSs), can be expressed in the nucleus and then transferred into the mitochondria, significantly reducing the amount of mutated mtDNA [102]. In these applications, REs specifically target mutated mtDNA sequences by recognizing unique nucleotide changes at the mutation site, thus avoiding cleavage of wild‐type mtDNA. This specificity ensures that only mutant mtDNA is degraded while leaving normal mtDNA intact. However, when the mutant and wild‐type sequences are highly similar, there is potential for off‐target effects, where normal mtDNA may also be cleaved [103].

Additionally, the introduction of the modified RE *Apa*LI, also with MTS and ATP5b 5′ and 3′ UTR sequences, into oocytes and single‐celled embryos significantly reduced the amount of BALB mtDNA [103, 104]. These findings suggest that the delivery of engineered endonuclease genes to patients holds promise for reducing the amount of mtDNA mutations through RE recognition. However, the editing efficiency of REs in these applications varies, depending on the accessibility of the target sequences and the overall heteroplasmy of the cells. In some studies, REs have successfully reduced mutant mtDNA levels by over 50%, leading to significant functional improvements in mitochondrial activity [102].

In conclusion, RE only recognizes limited sequences and cannot address the repair needs for extensive mitochondrial gene mutations, limiting the application of gene editing technology in mitochondria [100].

##### 5.2.1.2 Zinc Finger Nuclease

The limitations of RE have been circumvented by the advent of programmable nuclease technology. ZFN is a technology consisting of two components: a zinc finger DNA‐binding domain and a restriction nuclease *Fok*I domain. The DNA‐binding domain is made up of three groups of zinc finger proteins, each of which is composed of approximately 30 amino acids connected to a single zinc atom and linked to 3 base pairs of DNA. The *Fok*I domain, responsible for cutting DNA and causing fragment insertion or frameshift mutations of the target genes, is nonspecific and only functions as a dimer when both 5′ to 3′ and 3′ to 5′ domains are present [105]. When a ZFN binds to its target DNA sequence, the nuclease domain cleaves the DNA, creating a DSB at the site of binding. This DSB can then be repaired by one of two mechanisms: NHEJ or homology‐directed repair (HDR) [106]. NHEJ is an error‐prone repair pathway that often results in the insertion or deletion of a few nucleotides at the site of the DSB, which can disrupt the function of the targeted gene. HDR, on the other hand, uses a template DNA molecule to repair the DSB, allowing precise changes to be made to the targeted DNA sequence [106, 107]. This technology has been widely used to add, modify, or delete genes in nuclear genomes [108, 109]. In mtDNA, however, the absence of NHEJ and HDR pathways means that ZFN‐induced DSBs cannot be repaired as they are in nuclear genomes. Instead, DSBs in mtDNA often result in the degradation of the damaged DNA fragments [110]. While this degradation process can be useful for reducing the proportion of mutated mtDNA—thus potentially alleviating mitochondrial diseases caused by heteroplasmy—it also limits the precision of ZFN‐mediated mtDNA editing. Unlike nuclear editing, where DSBs can be used to introduce precise modifications, in mitochondria, DSBs primarily lead to the loss of mtDNA [110]. This dual role of ZFN‐induced DSBs in mitochondria—promoting the elimination of mutant mtDNA while preventing precise gene correction—presents both opportunities and challenges for therapeutic applications.

A major limitation of ZFNs in their application is the intricate protein–DNA interactions involved in the formation of a nucleotide triplet via the binding of a single zinc finger protein. This complexity renders the design of ZFNs targeting specific DNA sequences more challenging compared with TALEN or clustered regularly interspaced short palindromic repeats (CRISPR)‐based gene editing tools. It implies that designing ZFNs requires more meticulous adjustments and optimization, as a single zinc finger protein can only bind to a nucleotide triplet. In contrast, TALEN and base editing technologies can achieve more flexible targeted editing of specific DNA sequences by adjusting their components, as they rely on sequence‐specific domains of DNA. To address this, researchers have used MTSs and nuclear export signals (NESs) to direct ZFNs to the mitochondria [111, 112]. Additionally, a pair of monomers targeting mtDNA has been designed, with one binding to the wild‐type sequence and the other to the mutation site [113, 114]. This approach enhances the specificity of ZFNs for mtDNA editing. However, despite these efforts, the homologous dimers of the nucleic acid enzyme *Fok*I can cut mtDNA, but this method is less efficient and prone to off‐target effects. Due to the lack of a NHEJ repair pathway for DNA DSBs in mitochondria, damaged DNA is degraded [110]. A single‐stranded ZFN protein with double *Fok*I has been designed to improve accuracy and efficiency, but it also has limitations, such as the inability to identify deletions of long fragments such as common deletion (CD). Additionally, the lack of dimer restriction allows this single‐stranded ZFN protein to retain active nuclease activity, potentially causing cytotoxicity [113].

To reduce the incidence of off‐target effects, researchers have utilized heterologous dimers instead of homologous dimers. They have also adjusted the order of components, including the NES and label protein, in a way that reduced mutual interference and increased stability [113]. This approach resulted in a fivefold increase in the proportion of wild‐type mtDNA, surpassing the efficiency of single ZFN proteins with double *Fok*I editing, which only achieved a twofold increase [111]. Additionally, this method allowed for the identification of CD, a deletion sequence, by using two monomers designed to recognize wild‐type sequences on either side of the deletion. When a deletion mutation occurs, the heterodimer *Fok*I is positioned close enough to carry out nuclease activity [115]. Overall, these modifications improved the precision and effectiveness of the ZFN tool in editing mtDNA.

One promising application of ZFNs is in the treatment of mitochondrial diseases, which are caused by mutations in mtDNA. Due to the high demand for mitochondria in certain tissues, such as muscle and brain cells, and the specific symptoms that occur when the mtDNA mutation reaches a certain proportion, researchers have selected cell models with mitochondrial mutations associated with these diseases for further experiments [111]. Using mitochondrial ZFN (mtZFN), a type of ZFN specifically designed for targeting mtDNA, researchers have successfully reduced the proportion of mutated mtDNA in these cells. However, they also found that mtZFN can cause a decrease in the number of normal mtDNA genes, and in cells with high levels of mtDNA mutations, it may trigger cell death [116]. To address these issues, researchers have tried to reduce the knockout efficiency and use iterative knockout strategies. They have also attempted to use hammerhead ribozyme tools to control the expression level of mtZFN [116]. In addition to genetic proof, mutant gene knockout has also been verified at the organelle and cell levels. Researchers have observed improvements in ATP production capacity and energy charge after modifying the mtZFN. The amounts of citric acid and aconitic acid, which are two important products in mitochondrial metabolism, have also been promoted, further illustrating the effectiveness of mtZFN [117]. Furthermore, in vivo studies were conducted to further verify the feasibility of mtZFNs. A mouse model with a specific mtDNA mutation was used for the experiment. After tail intravenous injection of the associated virus, mtZFN expression was confirmed in heart cells, and improvements in the ratio of mitochondrial respiratory energy generation in the cell energy supply were observed, indicating the validity of mtZFNs in vivo [111].

Overall, the application of mtZFN in the treatment of mitochondrial diseases holds great promise, but further research is needed to address potential issues with its use, such as the potential for unintended mutations and off‐target effects. However, the results thus far are encouraging and suggest that mtZFN could be a powerful tool for treating a wide range of mitochondrial diseases in the future.

##### 5.2.1.3 Transcription Activator‐Like Effector Nuclease

Unlike ZFNs, transcription activator‐like effectors (TALEs) are DNA‐binding proteins that are found in certain plant pathogenic bacteria of the genus Xanthomonas [117]. They are part of a type III secretion system used by these bacteria to infect host cells [118]. TALEs consist of a variable number of 33–34 amino acid repeats, where each repeat recognizes and binds to a single nucleotide of DNA [118]. The specificity of TALE‐DNA recognition relies on two key factors: the repeat variable diresidue, located at positions 12 and 13 of each repeat, and the presence of a thymine at the invariable N‐terminal and C‐terminal domains (N0 position) in the target DNA sequence [119, 120]. TALENs are chimeric proteins that combine the DNA‐binding domain of a TALE with the DNA‐cleaving domain of a RE, *Fok*I. The *Fok*I domain cleaves DNA only when it is dimerized, so two TALENs are needed to target a specific DNA sequence and introduce a DSB [119]. TALENs can be designed to target almost any DNA sequence, making them a versatile tool for genome editing [121]. By creating a DSB, TALEN triggers the cell's DNA repair mechanisms, which can then be harnessed to introduce specific changes to the DNA sequence [122, 123]. For example, if a small piece of DNA with the desired mutation is introduced along with the TALEN, the cell's repair mechanisms may use that piece of DNA as a template to repair the DSB, resulting in the insertion of the desired mutation into the DNA [122].

In proof‐of‐concept studies, TALENs have demonstrated efficiency and accuracy in vitro, employing mitochondrial disease cell models (cybrids) and patient‐derived induced pluripotent stem cells (iPSCs) [117]. In cybrid models, the transient transfection of TALENs resulted in a significant alteration of heteroplasmy, favoring the presence of wild‐type mtDNA [124]. This was subsequently accompanied by the restoration of mtDNA copy numbers. Notably, these changes in mutant levels remained consistent over time and contributed to enhancements in mitochondrial function [117, 124, 125]. In vivo trials utilizing either systemic or localized administration of TALENs through adeno‐associated virus (AAV) vectors with muscle‐targeting properties have provided additional confirmation of their potential [126]. The application of a TALEN designed for the m.5024C>T mutation in mt‐tRNA^Ala^, delivered via an AAV9 vector to skeletal muscle, heart, and liver, led to the precise removal of mtDNA and the restoration of normal levels of mt‐tRNA [Ala] [127]. These experiments have not only successfully reduced mutant mtDNA levels but also led to molecular and physiological rescue, including the stabilization of mitochondrial tRNA levels and the recovery of mitochondrial metabolites. Importantly, mitoTALENs have demonstrated minimal off‐target effects, with a high degree of specificity for mtDNA and negligible impact on the nuclear genome [111, 128]. Consequently, TALENs represent a promising future therapeutic platform for individuals afflicted with mitochondrial genetic diseases, with ongoing research efforts aimed at refining delivery methods, enhancing specificity and ensuring the safety of TALEN‐based treatments for clinical applications.

#### 5.2.2 Mitochondrial Gene Editing Technology with Base Editors

**FIGURE 4:**
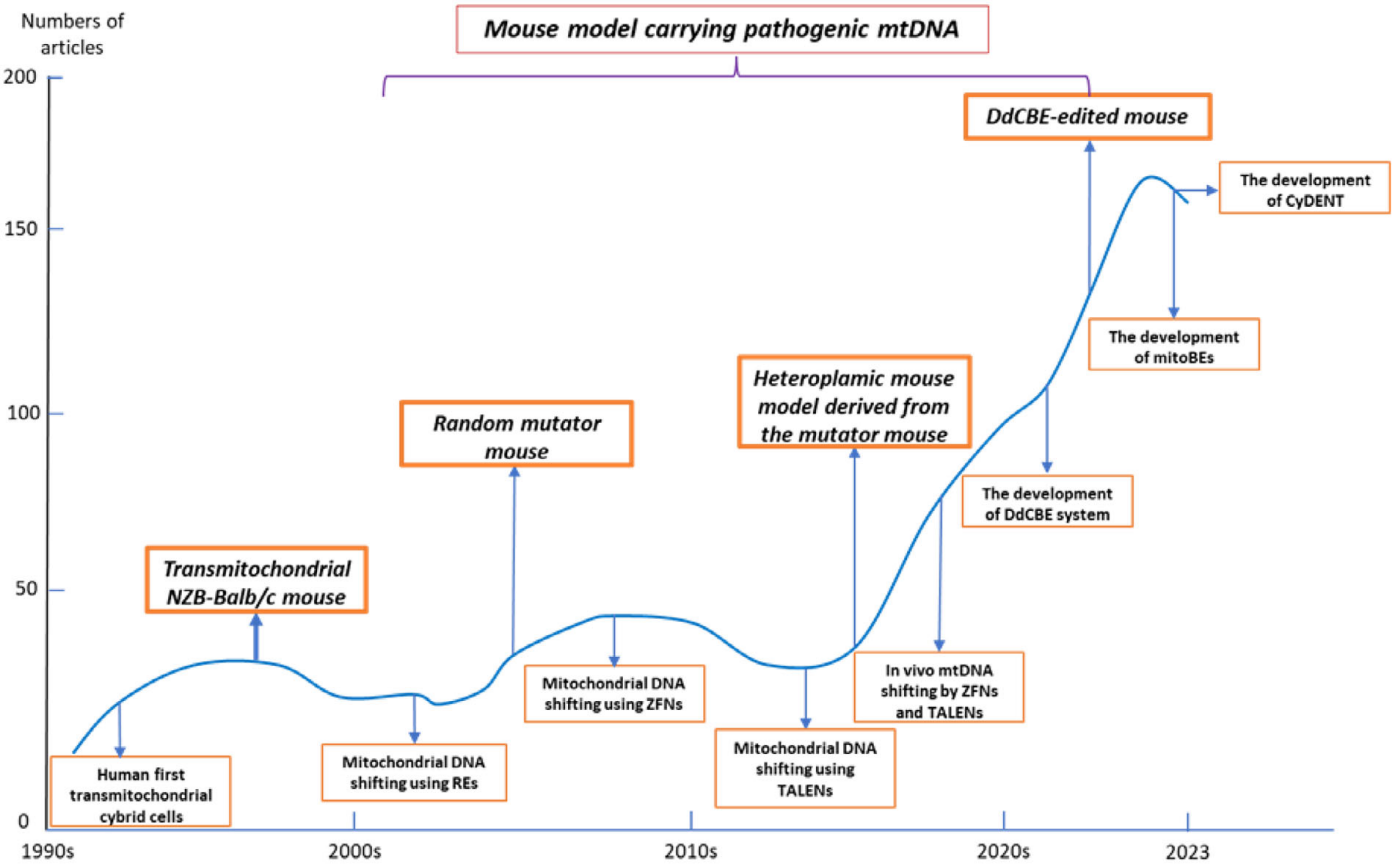
The development history of mtDNA editing: The curve chart presented herein delineates a comprehensive overview of annual research publications spanning the past three decades in the domain of mitochondrial gene editing. Accompanying this visual representation are annotations highlighting key breakthroughs that have significantly influenced the advancement of this field. Furthermore, a specific emphasis has been placed on the meticulous development of animal models, enriching our understanding of mitochondrial gene editing's practical applications. mitoBEs, mitochondrial DNA base editors; DdCBE, DddA‐derived cytosine base editor; mitoRE, mitochondrially targeted restriction endonuclease; mitoTALEN; mitochondrially targeted transcription activator‐like effector nuclease; mtZFN, mitochondrially targeted zinc finger nuclease.

Mitochondrial gene editing technologies utilizing nucleases face significant limitations in introducing specific de novo nucleotide changes in mtDNA, severely constraining our capacity to create accurate models of mitochondrial disorders. Additionally, these methods struggle to effectively target homoplasmic mtDNA mutations, further limiting their applicability in disease modeling and therapeutic interventions. Therefore, the advent of mitochondrial gene editing technology with base editors has become increasingly important. The utilization of CRISPR/CRISPR‐associated protein (Cas)9 system for editing mtDNA has demonstrated potential in correcting pathogenic mutations associated with mitochondrial diseases. However, the challenge lies in delivering the CRISPR/Cas9 system to mitochondria [129], requiring MTSs to specifically target the Cas9 protein. In the past 3 years, the development of DdCBE technology [130] has opened avenues for various base editing techniques, including TALE‐linked deaminase (TALED) [131], mitoBEs [132], and cytidine deaminase–exonuclease–nickase–TALE (CyDENT) [133]. These advancements bring new hope for mitochondrial gene editing, paving the way for potential clinical trials (Figure 4) (Table 3).

**TABLE 3:** Comparison and development of mtDNA editing technologies.

Application	RE	mtZFN	mitoTALEN	CRISPR/Cas9	DdCBE	mitoBEs	CyDENT
Interaction	Protein–DNA	Protein–DNA	Protein–DNA	RNA–DNA	Protein–DNA	Protein–DNA	Protein–DNA
Edit element	RE	*Fok*I	*Fok*I	Cas9	DddA	TALE‐TadA	TALE
Correction of mtDNA point mutations	Cutting the phosphodiester bonds	Heteroplasmic	Heteroplasmic	Not detected	(C‐to‐T) & (A‐to‐G)	(A‐to‐G) & (C‐to‐T)	Maybe all kinds of base editing
Correction of mtDNA	Yes	Yes	Yes	No	No	No	No
Correction of mtDNA deletions	No	Yes	Yes	No	No	No	No
Off‐target editing of mtDNA	High	High	High	Unknown	Low	Low	Low
Off‐target editing of nuclear DNA	High	Unknown	Unknown	Low	Relatively high	Low	Low
Therapeutic application	No	Potentially	Potentially	No	Potentially	Potentially	Potentially
AAV‐compatibility	No	One virus	Two viruses	One virus	Two viruses	Two viruses	Two virus
Advantage	Small	Easy to design	Easy to design	Highly specific	Highly specific	Highly specific	Highly specific

Abbreviations: AAV, adeno‐associated virus; CRISPR/Cas9, clustered regularly interspaced short palindromic repeats/CRISPR‐associated protein 9; CyDENT, cytosine deamination editors for mitochondrial DNA; DdCBE, DddA‐derived cytosine base editor; DddA, double‐stranded DNA‐specific cytidine deaminase toxin; mitoBEs, mitochondrial base editors; mitoTALEN, mitochondrial‐targeted transcription activator‐like effector nuclease; mtZFN, mitochondrial‐targeted zinc finger nuclease; RE, restriction endonuclease; TadA, tRNA adenosine deaminase; TALE, transcription activator‐like effector.

##### 5.2.2.1 CRISPR/Cas System

The CRISPR/Cas system, originally derived from bacterial immune defense mechanisms, has rapidly evolved into a versatile tool for genome editing, with significant implications for the treatment of human diseases. By utilizing the Cas9 protein, which induces DSBs at targeted genomic loci guided by a single‐guide RNA (sgRNA), CRISPR/Cas enables the precise modification of genes [134]. This system has been successfully applied in various fields, including cancer, genetic disorders, and infectious diseases. In particular, CRISPR/Cas has shown promise in cancer therapy, where it is used for gene knockout, activation, or correction to inhibit oncogenes or restore the function of tumor suppressor genes. Furthermore, CRISPR/Cas has been explored in the treatment of hereditary diseases such as Duchenne muscular dystrophy [135], sickle cell anemia [136], and cystic fibrosis [137], where it facilitates the correction of disease‐causing mutations. Ongoing clinical trials are investigating the therapeutic potential of CRISPR/Cas in treating hematological disorders, such as beta‐thalassemia [138] and sickle cell disease [139], by editing hematopoietic stem cells to correct the underlying genetic defects. Beyond genetic diseases, CRISPR/Cas is being evaluated for its efficacy in combating viral infections, including HIV [140] and hepatitis B [141], where it has been used to target and disrupt viral genomes. Despite these advances, the clinical application of CRISPR/Cas still faces challenges, including off‐target effects and delivery efficiency. However, advancements in delivery systems and the development of high‐fidelity Cas9 variants [142] and improved specificity tools [143] continue to improve its precision and safety, making CRISPR/Cas a revolutionary tool in the field of gene therapy.

In 2015, the application of the CRISPR/Cas9 system to mitochondrial gene editing was initially reported [144]; however, the findings were met with controversy, primarily due to the inability of mitochondria to import guide RNAs (gRNAs), which are essential for CRISPR/Cas9 function [129]. This technical challenge led to skepticism from other researchers, who questioned the feasibility of using CRISPR/Cas9 for mitochondrial editing in its traditional form. Despite this, the mechanism for introducing gRNA into mitochondria remains enigmatic. It has been proposed that a 20 ribonucleotide stem ring series derived from H1 RNA (RNase P enzyme RNA component) can effectively target RNA into mitochondria [145]. This method is believed to be effective for both noncoding RNA, such as tRNA, and mRNA encoding proteins, shedding light on the importation of gRNA into mitochondria within the CRISPR/Cas9 system [145]. The lack of NHEJ repair pathway in mitochondria, coupled with the existence of a homologous recombination repair pathway [146], has led to attempts and successes in inserting DNA sequences into mtDNA in various organisms [129, 147]. Overcoming challenges such as membrane potential, pH, and bacteriophage phagocytosis, foreign DNA has been successfully introduced into yeast mitochondria using various methods, including biolistic bombardment, electroporation, and complex formations [148]. Researchers utilized a MTS to modify the AAV capsid VP2, successfully facilitating the transportation of the ND4 gene into mitochondria [146]. Additionally, various other delivery systems for drugs or proteins targeted to mitochondria have been developed, including metal–organic frameworks, triphenylphosphonium, and cell‐penetrating poly(disulfide)s‐modified biodegradable silica nanoparticles [149, 150]. Interestingly, exogenous single‐stranded DNA (ssDNA) with a short homologous arm was accurately integrated into the targeting loci. This mutagenesis was stably transmitted to the F1 generation of zebrafish, indicating that ssDNA and gRNA can be transported into mitochondria without modification [151]. However, the existence of an endogenous mechanism for nucleic acid import into mammalian mitochondria, which is crucial for mitochondrial CRISPR/Cas9 gene editing, remains a subject of controversy.

##### 5.2.2.2 DdCBE System

Base editing technology represented by CRISPR/Cas has triggered a biotechnology revolution. CRISPR/Cas9 recognizes the target gene sequence through an artificially designed sgRNA. Under the guidance of sgRNA, the complex formed by CRISPR/Cas9 specifically binds to the target DNA by identifying the prospacer adjacent motif on the target DNA and cuts the target DNA [152, 153]. However, since the sgRNA cannot be efficiently imported into the mitochondria, CRISPR/Cas technology cannot be effectively used for mitochondrial genome editing [129].

An interbacterial deaminase‐like toxin was found to solve this problem. DddA, which is encoded by Burkholderia cenocepacia, can catalyze the deamination of cytidines within double‐stranded DNA (dsDNA) [130]. For detoxification, DddA_tox_ was split into two inactive halves, DddAtox‐N and DddAtox‐C. DddA_tox_ was reconstituted on predetermined DNA sites by fusing mitochondrially targeted TALE array proteins. To improve editing efficiency (to 22–27%), one copy of uracil glycosylase inhibitor (UGI) was added to the C‐terminus. Thus, the DdCBE was created and successfully edited five mtDNA genes in HEK293T cells with efficiencies varying between 4.6 and 49% [154]. DdCBE technology has great potential to identify new possibilities in the field of mtDNA manipulation. This technology enables researchers to reverse engineer the mitochondrial genome in animal cells and finally correct various mutation pathogenic points in mtDNA [130].

Although the original publication about DdCBEs only successfully constructed a disease cell model carrying mtDNA pathogenic point mutations, within a record short year, researchers successfully constructed different animal models using this technology and verified the possibility of species transmission [155, 156, 157, 158]. The targeted mitochondrial gene, MT‐ND5 (ND5), was successfully edited by delivering DdCBE mRNA in mouse embryos, and the mutation was maintained throughout development and finally inherited for a generation (F1) [155]. Another study also showed that transgenic female founders were able to transmit the mutations to their offspring with different mutation loads [156]. At the same time, a Chinese team successfully designed the DdCBE vector for zebrafish and rats and found that the PiggyBac vector efficiently facilitated systemic mtDNA editing by cloning the DdCBE pair into the vector [157, 158]. By vein‐injecting DdCBEs into the mouse heart using AAV, the adult and neonatal mouse MT‐ND3 gene was edited at mtDNA position 9577 with an editing efficiency of 10–20% [159]. In human embryos, by injecting DdCBE mRNA into human embryos with three pronuclei (3PN), two pathogenic mutation sites, ND1 and TRNK (G3733A, G8363A), were edited, which suggested the possibility of pathogenic mtDNA mutation correction in the human early embryonic stage [160]. In addition, comparing human zygotes and two‐cell, four‐cell and eight‐cell embryos, the DdCBE editing efficiency was found to be much higher in eight‐cell embryos [161].

Aiming at some defects of the original DdCBE version, scientists have made various attempts to optimize it. For example, the splitting of DddA_tox_ was incompetent in gene delivery viral vectors with a small cargo size, which drove scientists to develop nontoxic, full‐length DddAtox variants to make monomeric DdCBEs (mDdCBEs) that finally achieved C‐T editing in cultured human cells via AAV. The author also found that transfecting mDdCBE‐encoding mRNA was better than plasmids at reducing the off‐target effect [162]. Suspecting that spontaneous assembly of the split DddAtox deaminase enzyme leads to off‐target activity, scientists engineered high‐fidelity DdCBEs. Using alanine to substitute amino acid residues at the interface between the split DddAtox halves, the resulting domains could take effect only if they linked TALE proteins at adjacent sites on DNA, which made the base editor much more efficient and precise [163].

The initial DdCBE is limited predominantly to T‐C targets [154], so researchers have tried various methods to eliminate these restrictions. Through rapid phage‐assisted continuous evolution and phage‐assisted noncontinuous evolution methods [164, 165], researchers directed evolution to wild‐type DddA by identifying the variants DddA6 and DddA11, with which the editing efficiency of mtDNA bases at the T‐C site was increased by approximately 4.3 times on average [166]. Among them, DddA11 could edit target sequences more widely and edited T‐C, A‐C, and C‐C sites more efficiently [166]. Meanwhile, a recent article reported that TALEDs, a new complex that combined the research results of tRNA adenine deaminase (TadA8e, engineered from *E. coli* TadA [167]) with a DdCBE, could induce targeted A‐G editing in human mitochondria, in which DddA variants function in cis or in trans with TadA8e [131]. In this article, the author designed monomeric TALEDs (mTALEDs), dimeric TALEDs (dTALEDs), and split TALEDs (sTALEDs) to flexibly edit different mtDNA sites. Furthermore, unlike DdCBEs, TALEDs catalyze base editing independent of a 5′‐TC motif and show no obvious cell or mtDNA genomic toxicity [131]. Considering that A‐G conversions could correct many more pathogenic mutations in human mtDNA [52], it is urgent and necessary to evolve the original DdCBE.

Notwithstanding the success of DdCBEs and their bright prospects, the off‐target effect of DdCBEs remains a problem. The genome‐wide off‐target analysis by two‐cell embryo injection (GOTI) system and whole‐genome sequencing (WGS) were used to evaluate the targeting effect of DdCBEs on mtDNA and nucDNA modification [168, 169]. It was found that the targeted editing of DdCBEs at two different sites (G12918A, C12336T) was less than 5% in the mitochondria. However, 1500 and 1000 single‐nucleotide variants were generated in the nuclear genome. These results suggest that the MTS cannot prevent DdCBEs from entering the nucleus, which leads to serious missed target effects during editing [170]. In addition, TAS array sequence (TAS)‐dependent and TAS‐independent off‐target mutations were found by using Detect seq (d U‐detection enabled by C‐T transition during sequencing) technology in the human HEK‐239T cell line [171]. In addition, it was found that the DdCBE tool interacts with the important protein CTCF (CCCTC binding factor), which maintains the three‐dimensional structure of the genome [171].

##### 5.2.2.3 mtDNA Base Editors

Due to their enormous potential, many researchers are studying various new types of base editors. Recently, researchers have developed a novel approach to achieve efficient base editing in mtDNA using the TALE system [132]. By combining TALE‐fused nickase and deaminase, they established a powerful single base editor called a mitoBE. MitoBEs enable precise editing of A‐G or C‐T changes with remarkable efficiency, a capability not fully achieved by the DddA system. Moreover, they possess the unique ability to selectively edit specific DNA strands in mitochondria. Importantly, WGS revealed minimal off‐target effects in both mitochondria and nuclei. MitoBEs are further encoded in circular RNA, enabling efficient and selective base editing in mtDNA strands. In a disease model, mitoBEs demonstrated successful correction of pathogenic mutations in mtDNA. In patient‐derived cells with LHON, mitoBEs achieved an editing efficiency of approximately 20% at the target site. Remarkably, the corrected cells exhibited increased ATP content and oxidative respiration levels, indicating the potential therapeutic value of mitoBEs in treating mitochondrial genetic diseases [132]. This groundbreaking research marks the first‐time pathogenic mutations in mitochondria have been successfully corrected through base editing, offering promising prospects for future mitochondrial genome editing therapies.

##### 5.2.2.4 Cytidine Deaminase–Exonuclease–Nickase–TALE

TALE‐based tools for editing nuclear and organellar DNA traditionally rely on dsDNA deaminases, which edit substrate bases on both DNA strands, thereby compromising editing precision [132]. However, a recent advancement introduces CyDENT, a CRISPR‐free, strand‐selective, modular base editor [133]. CyDENT comprises a pair of TALEs fused with a *Fok*I nickase, a single‐strand‐specific cytidine deaminase, and an exonuclease to generate single‐stranded DNA substrates for deamination. Scientists have demonstrated the efficacy of CyDENT in editing nuclear, mitochondrial, and chloroplast genomes. By engineering mtCyDENT constructs tailored for HEK293T cells, they incorporated a MTS and optimized expression elements [133]. Notably, leveraging a small peptide, γb, to recruit deaminase, exonuclease, and UGI to the TALE–*Fok*I nickase at specific mitochondrial sites, scientists achieved editing efficiencies of 14% with a strand specificity of 95%. Furthermore, by substituting the CyDENT deaminase with one favoring GC motifs, they achieved up to 20% mitochondrial base editing at sites previously inaccessible to other methods [133]. The modular design of CyDENT offers versatility in customizing base editors for various applications.

##### 5.2.2.5 Advantages and Prospects of Base Editors

**FIGURE 5:**
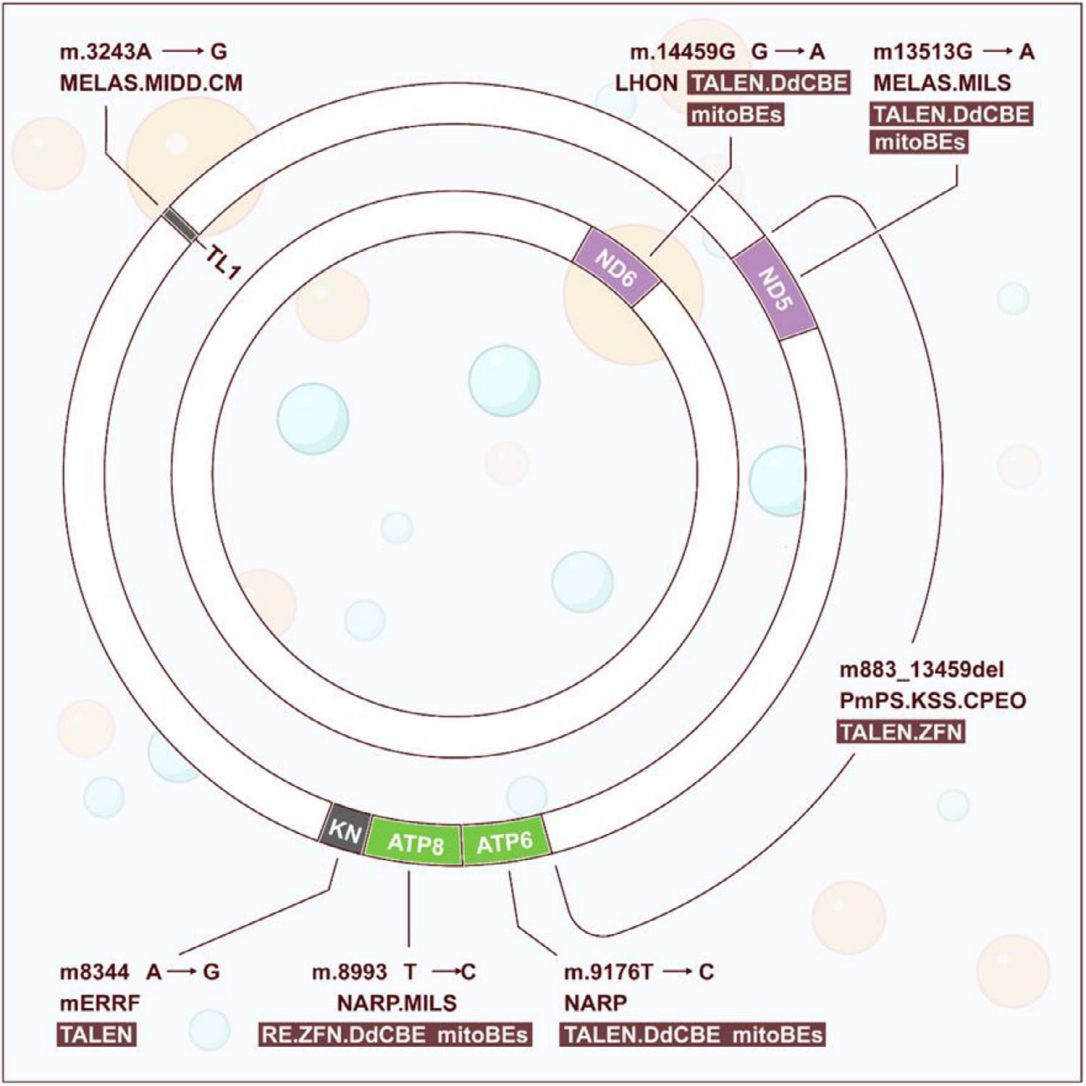
mtDNA mutations that have been or may be altered by gene editors: Summary of the mtDNA mutations corrected with available mitochondrial nucleases and in which tissues they have been successfully applied. ATP8/6, complex V subunits; CM, cardiomyopathy; CPEO, chronic progressive external ophthalmoplegia; DdCBE, DddA‐derived cytosine base editors; LHON, Leber hereditary optic neuropathy; MELAS, mitochondrial encephalomyopathy, lactic acidosis and stroke‐like episodes; MERRF, myoclonic epilepsy with ragged red fibers; MIDD, maternally inherited diabetes and deafness; MILS, maternally inherited Leigh syndrome; mitoBEs, mtDNA base editors; TALEN, targeted transcription activator‐like effector nuclease; ZFN, mitochondrially targeted zinc‐finger nuclease; NARP, neurogenic muscle weakness, ataxia, and retinitis pigmentosa; NCR, noncoding region; ND5, 6, complex I subunits; PMPS, Pearson marrow and pancreas syndrome.

The development of DNA base editors has significantly advanced our ability to precisely modify the mitochondrial genome, particularly through groundbreaking technologies, including DdCBEs and the emerging mitoBEs. DdCBEs, being the pioneers in organellar base editing, have brought about a transformative revolution in mitochondrial genome engineering. One of the key advantages of DdCBEs lies in their versatility and reliability. These tools have been meticulously engineered to precisely target and edit specific sequences within mtDNA [159, 161]. Their ability to induce single‐base changes in a programmable and controlled manner has opened up new possibilities for modeling and correcting mtDNA‐based disorders. Additionally, DdCBEs can be efficiently delivered into cells and animal models through various strategies, making them valuable assets for research and preclinical studies. MitoBEs, on the other hand, represent a promising advancement in the field of mitochondrial genome editing. These editors have the potential to expand the scope of mtDNA modifications by enabling the introduction of A‐G or C‐T changes in addition to canonical C‐T editing [132]. This broadens the applicability of mitochondrial base editing and enhances our ability to address a wider range of pathogenic mtDNA point mutations (Figure 5). Following the advancements in DNA base editors like DdCBEs and mitoBEs, the introduction of CyDENT marks another significant stride in the field of mitochondrial genome editing [133]. This novel tool demonstrates efficacy in targeting specific sequences within mtDNA, akin to DdCBEs, but with the added advantage of strand specificity. Moreover, CyDENT's modular nature allows for customization, offering versatility in addressing various mtDNA mutations and advancing research and preclinical studies. In essence, CyDENT represents a promising advancement that complements and enhances the capabilities of existing mitochondrial base editing technologies, ushering in new opportunities for precise modification of the mitochondrial genome.

Several trends are shaping the future of mitochondrial base editing. First, efforts to improve the specificity and precision of these editors are ongoing. Enhancing their ability to target specific sites within mtDNA while minimizing off‐target effects is crucial for their safe and effective application in gene therapy. Second, the development of alternative mtDNA editing technologies is gaining momentum. Researchers are exploring novel systems and strategies to complement the existing tools, allowing for more comprehensive mtDNA engineering. These innovations may include all‐protein DNA‐binding moieties and metagenomic approaches to discover unique deaminases with specific properties. Finally, the establishment of a reliable pathway for the import of nucleic acids into the mitochondrial matrix has the potential to democratize the field of mitochondrial genome engineering. This advancement could catalyze the development of technologies for basic research, disease modeling and gene therapy, making mitochondrial base editing more accessible and impactful.

In summary, DdCBEs and mitoBEs, as well as CyDENT, represent powerful tools for precise mitochondrial genome editing, offering hope for patients with mitochondrial diseases. Their continued refinement and the exploration of alternative technologies are paving the way for a future where mitochondrial base editing becomes a cornerstone of genetic medicine.

##### 5.2.2.6 Gene Correction of Nuclear‐Encoded Mitochondrial Genes

While mtDNA mutations are a well‐recognized cause of mitochondrial disease, the majority of mitochondrial proteins—more than 1000—are encoded by nucDNA [50]. Consequently, mutations in nuclear genes such as TK2, POLG, SURF1, and OPA1 underlie a significant fraction of mitochondrial disorders, including mtDNA depletion syndromes, LS, and dominant optic atrophy [173]. These mutations often impair mitochondrial biogenesis, replication, or membrane dynamics, and cannot be addressed by mtDNA‐directed therapies alone.

To correct these mutations, several nuclear gene therapy strategies are under development. AAV‐mediated gene replacement has shown particular promise. For instance, AAV9–TK2 delivery restored thymidine kinase 2 function and significantly extended survival and neuromuscular function in a mouse model of TK2 deficiency, paving the way for ongoing clinical trials (NCT04174105) [174]. Similarly, CRISPR–Cas9 editing has been used to repair SURF1 mutations in patient‐derived cells, restoring COX activity and suggesting potential for treating LS [175]. Another strategy employs antisense oligonucleotides (AONs) to selectively silence dominant‐negative alleles, such as those seen in OPA1‐associated optic atrophy, with promising results in iPSC models [176].

Despite these advances, challenges remain. AAV‐based delivery is limited by payload capacity and immunogenicity. Long‐term expression control, tissue‐specific targeting—especially in the brain and muscle—and avoiding off‐target editing effects are key hurdles. Moreover, due to the heterogeneity of nuclear gene mutations across patients, personalized vectors and editing strategies may be required. Still, recent progress supports the feasibility of nuclear genome correction as a viable therapeutic modality for a subset of mitochondrial diseases that are otherwise untreatable via mtDNA‐targeted approaches [177].

### 5.3 Mitochondrial Transplantation for Mitochondrial Diseases

Mitochondrial transplantation and replacement strategies have emerged as cutting‐edge approaches for treating mitochondrial diseases, particularly those caused by pathogenic mtDNA mutations [178]. One branch of these therapies involves mitochondrial replacement therapy (MRT), whose primary objective is to transfer the nuclear genetic material from a zygote or oocyte at risk of transmitting mitochondrial disease into an enucleated donor cell with healthy mitochondria. The two most prominent MRT techniques are pronuclear transfer (PNT) and maternal spindle transfer (MST) [179, 180, 181]. PNT involves transferring the pronuclei from a fertilized zygote into a donor zygote with normal mitochondria, whereas MST transfers the metaphase spindle from a mother's egg into an enucleated donor egg before fertilization [179, 180, 181]. Both methods allow the nuclear genome from the biological parents to be combined with healthy donor mitochondria, thereby preventing the maternal transmission of mtDNA disorders. While MRT is permitted in countries such as the United Kingdom under tightly regulated frameworks [182], it remains restricted in others, such as the United States, due to ethical concerns over germline modification and the creation of “three‐parent” offspring [182, 183].

In parallel, mitochondrial transplantation—the direct transfer of functional mitochondria into cells or tissues—has shown preclinical promise in restoring bioenergetic capacity in affected tissues [184]. However, successful implementation depends on multiple technical variables, including the method of mitochondrial isolation, source (autologous vs. allogenic), route of administration (e.g., microinjection, vesicle‐mediated transfer), and dosage [185]. One significant hurdle is maintaining mitochondrial viability postisolation, as mitochondria are extremely sensitive to oxidative stress and typically lose function within 24 h [185, 186]. The use of antioxidants such as Trolox can help preserve function, and vitrification methods have shown improved survival over traditional cryopreservation, though further optimization is needed [187]. Long‐term storage protocols remain undeveloped [187], forcing reliance on freshly isolated mitochondria. Another challenge is the immune response elicited by allogenic mitochondria or donor‐recipient mtDNA mismatches, potentially triggering inflammation or epigenetic instability. Haplotype matching and immunosuppressive strategies are being explored to mitigate these risks.

Furthermore, precise delivery methods must be tailored to specific tissue types to ensure mitochondrial uptake and functional integration. Although approaches such as direct microinjection and vesicle encapsulation have been studied, they can interfere with cellular processes and limit efficiency [178, 188]. An emerging strategy involves binding mitochondria to carrier proteins, which shows promise for improving tissue specificity but requires further development and safety validation [189].

In conclusion, mitochondrial replacement and transplantation therapies represent a promising, albeit complex, frontier in the treatment of mitochondrial diseases. While Phase I clinical trials for PNT are underway in the United Kingdom, the broader application of these technologies will depend on overcoming biological barriers such as mitochondrial storage, immune compatibility, and delivery efficiency. Simultaneously, ongoing bioethical discourse surrounding germline modification will continue to shape regulatory acceptance and clinical translation across regions.

## 6 Conclusions and Perspectives

Mitochondrial diseases represent a diverse and significant group of inherited disorders characterized by defects in mitochondrial function, often due to mutations in mtDNA [190]. These conditions are clinically heterogeneous—affecting multiple organs with varying severity—and their molecular mechanisms are exceedingly complex. Factors such as heteroplasmy, oxidative stress, and impaired mitochondrial quality control interact in a multifaceted manner, making genotype–phenotype correlations difficult to predict. This complexity has historically posed challenges for diagnosis and treatment, and indeed there are currently no curative or United States Food and Drug Administration‐approved disease‐modifying therapies for mitochondrial diseases [191, 192] (Table 4). Nevertheless, substantial progress in research over recent years has deepened our understanding of the pathogenesis of these disorders and highlighted their importance in biomedical research. This improved insight is laying critical groundwork for the development of targeted interventions. On the therapeutic front, emerging strategies are offering new hope. Advanced mitochondrial‐targeted gene therapy approaches have shown particular promise in preclinical models. For example, precision gene‐editing tools—including mtZFNs, mitoTALENs, and base editors such as DdCBE—can selectively alter or eliminate pathogenic mtDNA mutations. These technologies have demonstrated the ability to correct mitochondrial genetic defects in vitro and in vivo, effectively rescuing cellular disease phenotypes. Such advancements have instilled hope that true disease‐modifying treatments for mtDNA‐related conditions may soon become a reality. Notably, one of these tools (mitoTALEN) has already entered a Phase I clinical trial in patients, and a first‐in‐human trial of a base editor (DdCBE) is anticipated in the near future. In parallel, mitochondrial replacement techniques (e.g. MRT) are being explored to prevent the maternal transmission of pathogenic mtDNA variants. Beyond genetic therapies, other therapeutic avenues—including metabolic cofactors, small‐molecule approaches, and gene expression modulators—are under investigation as part of a comprehensive effort to manage these diseases.

**TABLE 4:** Therapeutic strategies for mitochondrial diseases mapped to pathogenic mechanisms and evidence levels.

Pathogenic mechanism	Therapeutic strategy	Representative examples	Evidence level
OXPHOS defects	Metabolic/small molecule therapy	Coenzyme Q10, idebenone, EPI‐743	Clinical
Heteroplasmy threshold	Gene editing	DdCBE, mitoBEs, TALEDs, CyDENT	Preclinical
mtDNA maintenance/repair deficiency	Mitochondrial replacement/transplantation	MST, PNT, mitochondrial transplantation	Clinical
Inflammation/immune axis	Immunomodulation/anti‐inflammatory agents	Corticosteroids, IL‐1 blockers	Clinical/preclinical

Abbreviations: CyDENT, cytosine deamination‐enabled nucleotide transporter; DdCBE, DddA‐derived cytosine base editor; mitoBEs, mitochondrial base editors; MST, maternal spindle transfer; mtDNA, mitochondrial DNA; OXPHOS, oxidative phosphorylation; PNT, pronuclear transfer; TALEDs, transcription activator‐like effector‐derived deaminases.

Given the multisystem nature of mitochondrial disorders, it is likely that a combination of interventions and supportive care will be required to address the full spectrum of clinical needs. In essence, current research is building a diverse therapeutic arsenal, from gene editing and MRT to pharmacological and dietary strategies, to tackle mitochondrial diseases from multiple angles. Despite this progress, formidable challenges remain before these advances can translate into widely effective treatments. One major hurdle is ensuring safety and targeted delivery for novel therapies. For instance, gene‐editing approaches must achieve high specificity to avoid off‐target effects in both the mitochondrial and nuclear genomes, and efficient delivery systems are needed to transport therapeutic molecules into patients’ mitochondria. Temporary reductions in mtDNA copy number or interference with mtDNA repair pathways during treatment could also have unintended deleterious consequences, underscoring the need for thorough preclinical safety evaluations. Additionally, the extreme genetic and clinical heterogeneity of mitochondrial diseases means that designing clinical trials and measuring therapeutic efficacy can be difficult. Many patients present unique combinations of symptoms, and reliable biomarkers for early diagnosis or disease monitoring are limited. This lack of specific biomarkers and the variability among patients have contributed to the current situation in which management is often largely supportive and symptom‐focused. Overcoming these translational obstacles—from improving biomarker availability to tailoring interventions for individual genetic profiles—is critical for moving experimental therapies from bench to bedside. Looking ahead, future research directions will need to address both scientific and clinical challenges to fully realize effective treatments for mitochondrial diseases.

On a fundamental level, continued in‐depth investigation of mitochondrial disease biology is required. Further elucidating the molecular pathogenesis—for example, how various mtDNA mutations and nuclear gene interactions lead to tissue‐specific pathology—will not only advance basic knowledge but also reveal new therapeutic targets. In parallel, efforts to develop improved diagnostic tools are essential. Identifying sensitive biomarkers for early detection and progression will enable timelier interventions and better tracking of treatment responses [190]. Therapeutically, ongoing innovation and refinement of interventions must continue. This includes optimizing gene‐editing systems for greater precision and safety, improving mitochondrial delivery methods, and exploring novel therapeutic platforms or drug candidates. Importantly, a multidisciplinary treatment strategy should be pursued: combining emerging disease‐targeted therapies with existing supportive measures and personalized care plans, in order to address both the underlying mtDNA defects and the immediate clinical needs of patients. Furthermore, robust clinical trial designs and international collaboration will be needed to surmount the hurdles posed by small patient populations and diverse phenotypes. As some interventions (like germline mitochondrial modifications) could have heritable effects, researchers and policymakers must also work together to establish clear ethical guidelines and safety frameworks for future clinical applications.

In summary, mitochondrial diseases remain highly significant yet challenging disorders due to their complexity, but the landscape is rapidly evolving. Advances in molecular genetics and biotechnology have opened new avenues for treatment—from gene editing to mitochondrial replacement—that hold genuine promise for altering the course of these conditions. At the same time, the necessity for continued research is evident: only through ongoing scientific inquiry, technological innovation, and clinical rigor can we overcome the remaining obstacles. By addressing the disease mechanisms more completely, improving early diagnosis, and developing safe and effective therapies, the field of mitochondrial disease research is steadily moving toward a future in which these once intractable disorders can be better managed, and possibly even prevented, thereby offering renewed hope to patients and families worldwide.

## Author Contributions

J.M., P.D., and J.G. drafted and conceived the initial manuscript. C.G. and C.Z. provided the essential assistant for our final manuscript. J.M. and Z.J. drew the figures and arranged the tables. J.G. and C.Z. revised the manuscript. All authors have read and approved the article.

## Ethics Statement

The authors have nothing to report.

## Conflicts of Interest

The authors declare no conflicts of interest.

## Data Availability

The data that support the findings of this study are available from the corresponding author upon reasonable request.
